# Research priorities to reduce the impact of musculoskeletal disorders: a priority setting exercise with the child health and nutrition research initiative method

**DOI:** 10.1016/S2665-9913(22)00136-9

**Published:** 2022-07-27

**Authors:** Zoe Paskins, Clare E Farmer, Fay Manning, David A Andersson, Tim Barlow, Felicity L Bishop, Christopher A Brown, Amanda Clark, Emma M Clark, Debra Dulake, Malvika Gulati, Christine L Le Maitre, Richard K Jones, John Loughlin, Deborah J Mason, Maura McCarron, Neil L Millar, Hemant Pandit, George Peat, Stephen M Richardson, Emma J Salt, E Jane Taylor, Linda Troeberg, Ruth K Wilcox, Elspeth Wise, Colin Wilkinson, Fiona E Watt, Musculoskeletal Disorders Research Advisory Group Versus Arthritis

**Affiliations:** a Primary Care Centre Versus Arthritis, Keele University, Keele, UK; b School of Medicine, Keele University, Keele, UK; c College of Medicine and Health, University of Exeter, Exeter, UK; d Versus Arthritis, Chesterfield, UK; e Wolfson CARD, Institute of Psychiatry, Psychology & Neuroscience, King's College London, London, UK; f Department of Orthopaedics, Wrightington Hospital, Wigan, UK; g School of Psychology, University of Southampton, Southampton, UK; h Department of Psychology, University of Liverpool, Liverpool, UK; i Bristol Medical School, University of Bristol, UK; j Nuffield Department of Orthopaedics, Rheumatology and Musculoskeletal Sciences, University of Oxford, Oxford, UK; k Centre for Health Sciences Research, University of Salford, Manchester, UK; l Biomedical Sciences Research Centre, Sheffield Hallam University, Sheffield, UK; m Biosciences Institute, Newcastle University, Newcastle upon Tyne, UK; n School of Biosciences, Cardiff University, Cardiff, UK; o Department of Rheumatology, Belfast Health and Social Care Trust, Belfast, UK; p Institute of Infection, Immunity and Inflammation, College of Medicine, Veterinary and Life Sciences, University of Glasgow, Glasgow, UK; q Leeds Institute of Rheumatic and Musculoskeletal Medicine, University of Leeds, Leeds, UK; r Institute of Medical and Biological Engineering, University of Leeds, Leeds, UK; s Division of Cell Matrix Biology and Regenerative Medicine, School of Biological Sciences, Faculty of Biology, Medicine and Health, Manchester Academic Health Sciences Centre, University of Manchester, Manchester, UK; t University Hospitals of Derby and Burton NHS Foundation Trust, Derby, UK; u Norwich Medical School, University of East Anglia, Norwich, UK; v Talbot Medical Centre, South Shields & Primary Care Rheumatology and Musculoskeletal Medicine Society, South Shields, UK; w Department of Immunology and Inflammation, Imperial College London, London, UK

## Abstract

Involving research users in setting priorities for research is essential to ensure the outcomes are patient-centred and maximise its value and impact. The Musculoskeletal Disorders Research Advisory Group Versus Arthritis led a research priority setting exercise across musculoskeletal disorders. The Child Health and Nutrition Research Initiative (CHNRI) method of setting research priorities with a range of stakeholders was used, involving four stages and two surveys, to: (1) gather research uncertainties, (2) consolidate these, (3) score uncertainties against importance and impact, and (4) analyse scoring for prioritisation. 213 people responded to the first survey and 285 people to the second, representing clinicians, researchers, and people with musculoskeletal disorders. Key priorities included developing and testing new treatments, better treatment targeting, early diagnosis, prevention, and better understanding and management of pain, with an emphasis on understanding underpinning mechanisms. We present a call to action to researchers and funders to target these priorities.

## Introduction

Rheumatic musculoskeletal diseases include two broad areas: inflammatory rheumatic musculoskeletal diseases (inflammatory arthritides, autoimmune diseases, and multisystem diseases) and other musculoskeletal disorders. These other disorders include a range of short-term and long-term conditions that affect the musculoskeletal system, including many highly prevalent disorders such as osteoarthritis, osteoporosis, and back pain.^1^, ^2^ These disorders are characterised by pain and impaired physical function, often increasing the risk of immobility, obesity, other comorbidities including chronic physical and mental health conditions, some vascular conditions, and all-cause mortality.^3^, ^4^ Musculoskeletal disorders affect an estimated 20 million people across the UK, with one in five people consulting primary care for them annually.^5^, ^6^ Musculoskeletal disorders account for more than 22% of the total burden of ill health in the UK and the third largest area of National Health Service (NHS) programme spending (£4·7 billion), with substantial costs including total joint replacement and other forms of orthopaedic surgery.^5^ Furthermore, the societal impact is great. Musculoskeletal disorders are the leading cause of disability at work, sickness absence from work, and presenteeism, resulting in lost productivity as high as 2% of gross domestic product.^7^ As the incidence of many non-inflammatory musculoskeletal disorders increases with age, and population profiles in high-income countries are becoming older, the prevalence of these disorders is set to increase. In recognition of its impact and unmet needs, several musculoskeletal disorders, including osteoarthritis and back pain, have been designated as serious diseases by the US Food and Drug Administration.^8^ Recognising the public health importance of musculoskeletal health across the course of life, a new multisector 5-year prevention framework was launched in England in 2019.^9^

Research into musculoskeletal disorders has been shown to produce a 25% economic return on investment; considerably greater than the rate of return for cancer research (10·0%) and the UK Government minimum threshold for investment (3·5%).^5^, ^10^ However, although musculoskeletal conditions accounted for 11·5% of disability-adjusted life-years in the UK in 2017, they received only 4·5% of research funding in 2014.^5^

In 2019, Versus Arthritis, the largest musculoskeletal charity in the UK, convened a Musculoskeletal Disorders Research Advisory Group with the long-term aim of improving quality and impact of musculoskeletal disorders research.^11^

This group considered the current state of knowledge and research into musculoskeletal disorders and the current evidence for how research activity in this area might be prioritised by governments and funding agencies. Although more than 20 research priority publications in musculoskeletal disorders were identified by the group, these either focused on individual musculoskeletal disorders,^12^, ^13^, ^14^, ^15^, ^16^, ^17^, ^18^, ^19^, ^20^, ^21^, ^22^, ^23^, ^24^, ^25^ causes,^26^, ^27^ or treatments.^14^, ^16^, ^25^, ^28^, ^29^ Only one publication addressed a wide range of musculoskeletal disorder research priorities, but was not derived using formal prioritisation methods.^30^ Some priorities were informed by either the views of experts or patients alone.^21^, ^22^, ^23^, ^26^ Involving all relevant research users in priority setting, including people with lived experience, is essential to ensure research outcomes are patient-centred, relevant, have a high likelihood of resulting in patient benefit, increase research value and impact, and reduce research waste.^31^ A scoping review of priority setting exercises in rheumatic disorders identified that most published priorities focused on treatments rather than economic impact, implementation, or early discovery science.^32^ Limitations identified by this review included a paucity of robust methods, research questions that were not answerable, and an absence of implementation strategy.^32^ In summary, several research priorities have been identified, but none comprehensively address the full range of musculoskeletal disorders across all stages of research and involving all stakeholders. To address this gap, the Musculoskeletal Disorders Research Advisory Group Versus Arthritis aimed to conduct a formal research prioritisation setting exercise across musculoskeletal disorders.

## Methods

### Project oversight, aims, and scope

The Musculoskeletal Disorders Research Advisory Group Versus Arthritis (henceforth referred to as ‘the group’) designed, led, and contributed to this research; the group has 26 members including public contributors with lived experience of a range of musculoskeletal disorders, researchers, and health-care professionals. The group and its membership are facilitated by the charity. Among clinician and researcher members, there is a wide representation of clinical specialties (general practitioners, orthopaedic surgeons, physiotherapists, and rheumatologists), of subspeciality interest and research disciplines (clinical and applied health services researchers, discovery scientists, and epidemiologists). The group is committed to diversity, with representation of varying gender, ethnicity, age, geographical location, and stage of career. The group determined the purpose and remit of the priority setting exercise, including the definition of the population, audience, and timeline of interest (panel). The long-term goal of the research prioritisation exercise was to improve the quality and impact of research, which seeks to develop our understanding and management of musculoskeletal disorders.

Panel **Context, purpose, and remit of musculoskeletal research priority setting exercise**

**Long-term goal**

To improve the quality and impact of research which seeks to develop our understanding and management of musculoskeletal disorders. Specifically:

• to enhance prevention, early detection, and treatment and care of musculoskeletal disorders
• to improve the quality of life and wellbeing of those with musculoskeletal disorders
• to reduce personal, social, and economic burden of musculoskeletal disorders

**Population of interest**

• People (aged 18 years or older) with, or at risk of, musculoskeletal disorders, their families, carers, and health-care providers
• Musculoskeletal disorders, defined as: osteoarthritis, crystal disease such as gout, primary and secondary causes of musculoskeletal pain including regional and widespread pain (such as back pain, shoulder pain, and tendinopathy; other regional pain syndromes; and fibromyalgia), hypermobility, metabolic bone disorders (such as osteoporosis and rare diseases), and musculoskeletal injuries caused by acute traumatic events
• Participants and research questions within the UK were identified as the main focus, although the findings could well have relevance beyond the UK

**Timeframe for impact**

Both within and beyond 3 years (0–3 years [short term], >3 years [longer term])

**Research domains identified a priori**

*Diagnosis and impact (diagnosis)*

• Achieving an early and accurate diagnosis
• Measuring the true impact of musculoskeletal disorders on individuals and on society
• Maximising the potential of electronic health records

*Living well with musculoskeletal disorders (living well)*

• Improving self-management and support in the home or community
• Improving any aspect of health-care treatment

*Mechanisms of disease (mechanisms)*

• Understanding the causes and development of musculoskeletal disorders
• Disease processes shared between disorders

*Successful translation (translation)*

• Ensuring that research-proven tests, treatments, and approaches are routinely available in clinical practice
• Enabling discoveries to move from the laboratory to the clinic, towards patient benefit

**Audience**

• People with musculoskeletal disorders
• Researchers of all stages
• Health-care professionals
• Funders and other agencies who influence the funding and research strategy of musculoskeletal disorders
• Industry, such as pharmaceutical and medical technology companies

### Overview of methods

**Figure 1:**
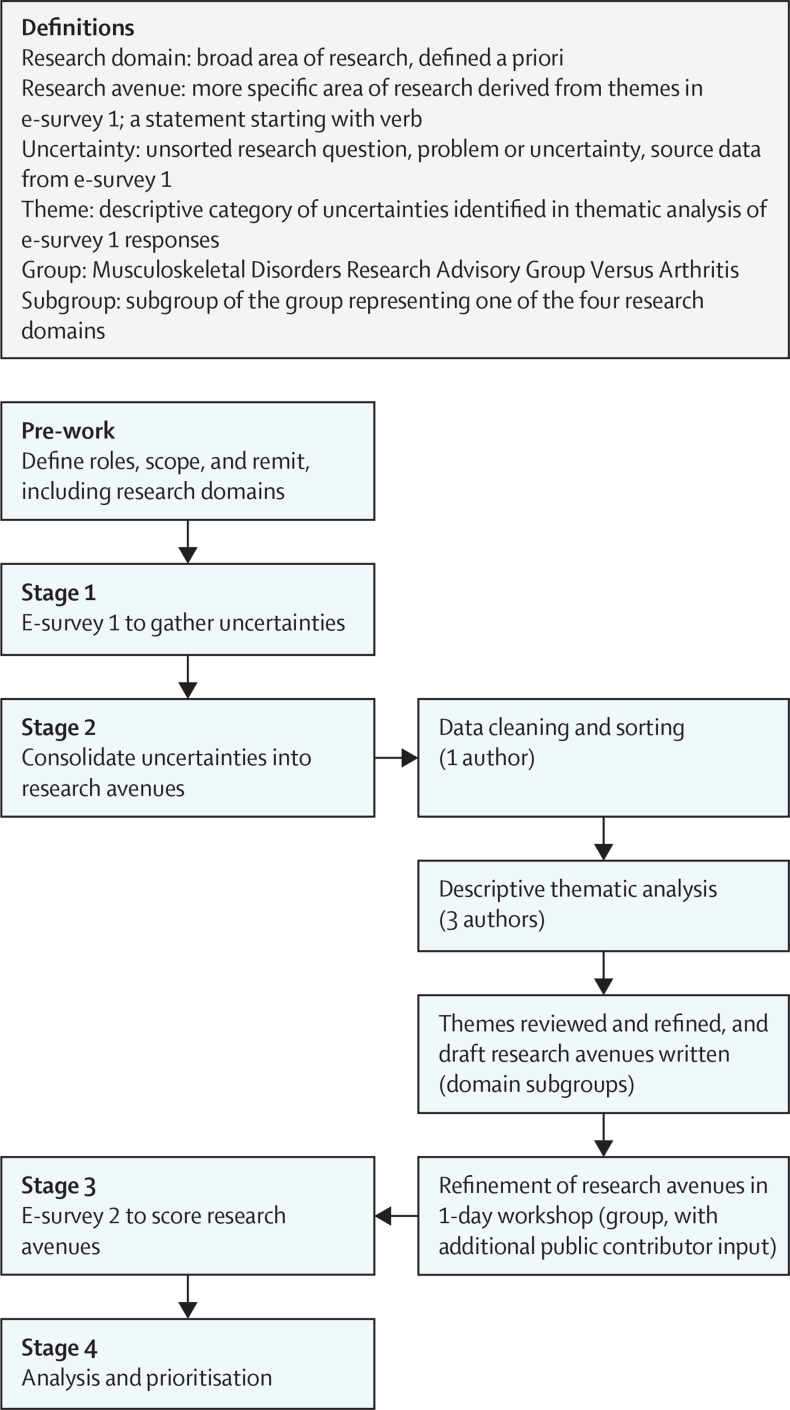
Overview of methods

The group considered and compared a range of prioritisation methods and selected the Child Health and Nutrition Research Initiative (CHNRI) method of priority setting as it enabled a range of stakeholder perspectives to be incorporated; could consider discovery science, clinical, and applied health services research questions together; and would incorporate objective scoring to enable ranking.^33^, ^34^ In addition to the first step of establishing scope and purpose, the CHNRI method involves four main stages to: (1) gather research uncertainties, (2) consolidate uncertainties, (3) score uncertainties with agreed criteria, and (4) analyse scoring for prioritisation (figure 1). The detailed methods, including design and distribution of two surveys (for stages 1 and 3), agreed scoring criteria, method of analysis and ranking, were all predefined by the group in advance in a published protocol.^35^ In a modification to the originally described CHNRI method, we involved all stakeholders, including public contributors, at every stage, as has been done previously.^36^, ^37^ The CHNRI uses specific terms to describe suggested areas of research, which we adopted (figure 1). Briefly, research domains are defined as broad areas of research, whereas research avenues are more specific areas within a research domain, which might align to research funder calls and could include several more specific research questions. In the CHNRI process, research avenues are identified, scored, and prioritised. Research domains were identified a priori (panel) on the basis of agreed importance, potential impact, and research unmet need.^35^ These were: diagnosis and impact; living well with musculoskeletal disorders; mechanisms of disease; and successful translation. The group appointed a lead with relevant experience for four subgroups representing each research domain; a public contributor was appointed to lead the living well subgroup (EC for diagnosis, CW for living well, CLLM for mechanisms, and DJM for translation; full subgroup membership is detailed in Contributor's statement). The details of the four stages are presented in the following sections and in further detail in the published protocol.^35^ Ethical approval for the research prioritisation exercise was given by a University Research Ethics Committee (Medical Sciences Division, University of Oxford, Oxford, UK; R71769/RE001).

### Stage 1: gathering research uncertainties (e-survey 1)

An electronic survey (e-survey 1) was developed, which asked for important uncertainties or unanswered questions (hereafter referred to as uncertainties) that could be answered by research, to be distributed to our audience (as defined in panel). Participants gave electronic consent to participate following provision of information about the exercise. E-survey 1 asked for important uncertainties within each of the four research domains, but participants could also enter uncertainties in an uncategorised section of the e-survey (appendix 2 pp 2–4). E-survey 1 also included questions on which domain was perceived as most important, perceived barriers to research (to be reported separately), demographics, and, where relevant, research experience and interests, and type of employment. E-survey 1 was distributed, aiming to reach different stakeholders in each of these predefined audience groups (panel), via: Versus Arthritis existing mailing lists and newsletters for researchers, health professionals, and lay volunteer research partners; web pages and social media (Versus Arthritis, other charities and organisations, and patient groups); and professional networks identified and accessed by the group (appendix 2 p 12). Group members were able to complete e-survey 1, which remained open for a 6-week period between Nov 5, 2020, and Jan 17, 2021.

### Stage 2: consolidating uncertainties and generating research avenues

The process of consolidating the uncertainties into avenues is detailed in figure 1. First, the uncertainties from e-survey 1 were separated from the responder characteristics (by CEF). The anonymised uncertainties then underwent data cleaning (by FM) to (1) separate text entries containing multiple entries to individual numbered uncertainties, (2) remove out-of-scope uncertainties (eg, outside of our population of interest as defined in the panel), (3) allocate uncategorised uncertainties entered in the survey's ‘other’ section to a research domain, and (4) reclassify uncertainties to different domains, where considered appropriate. Second, the uncertainties within each domain were coded and grouped into themes with descriptive thematic analysis (by FM). This process was achieved by familiarisation with the data, coding the whole dataset, and looking for themes across the coded data, in an iterative process with discussion between FM, FEW, and ZP.^35^ FM produced a draft list of themes for each research domain. Each domain subgroup held one to two meetings in which the draft list of themes was discussed and refined, with reference to the source data (e-survey 1 uncertainties). In these meetings, the domain sub-groups reviewed, edited, and agreed themes and subsequently drafted potential research avenues corresponding to each theme. The research avenues were drafted to start with a verb (eg, evaluate, determine, or investigate).

A one-day group workshop was convened to review the themes including any duplication, consider the appropriateness of the research domains derived a priori, and to refine the research avenue wording. In refining the wording, attention was paid to consistency between the domains and balance between research avenues to ensure they were not too broad or specific and used similar (lay) terminology. Finally, the research avenues underwent further review by an additional group of lay volunteer research partners, involved in Versus Arthritis but independent of this group, which resulted in further edits to improve readability. These were presented to, and agreed, with the group by email.

### Stage 3: scoring the research avenues (e-survey 2)

The consolidated research avenues were scored with a second e-survey (e-survey 2). The original CHNRI method suggested that each research avenue should be scored against five criteria.^36^ Subsequently, researchers adapted the number of criteria (more and less than five), and also introduced their own criteria.^38^ The group reviewed a range of criteria previously associated with the CHNRI method^36^, ^38^ and initially identified importance, impact, and feasibility as the most important criteria. Equity was considered a cross-cutting criterion that should be reflected throughout the process and criteria, but was not specifically scored. Following feedback from public contributors, two criteria, importance and impact, were felt sufficient, minimised the length and burden of e-survey 2, and maximised its accessibility to intended stakeholders.^35^

In the survey, which was co-designed by the group and pilot tested by public contributors, the list of research avenues was presented with two questions representing the scoring criteria for importance (“Will this research lead to important new knowledge?”) and impact (“Will this research make a difference and lead to impact?”). Respondents were asked to score to what extent each research avenue met these criteria, using a numeric rating scale, where 1 equated to ‘not likely’ and 10 equated to ‘extremely likely’. An “I am unsure” option was also available. Answers could also be missed and there was an option to save and return. It was made clear that each respondent should answer based on their own knowledge and perspective. Optional additional questions on age, sex and ethnicity, employment, and interests, similar to e-survey 1, were included at the end of the survey (for the full survey text see appendix 2 p 5–11). Each respondent received the questions in a random order, meaning that partial completion of the e-survey would not result in some questions having many more responses than others, preventing bias in scoring.

E-survey 2 was distributed to participants from the first survey who gave their consent to further email contact as well as being advertised more broadly, through similar channels to the first survey (appendix 2 p 12). Group members were excluded from participating in the e-survey 2. E-survey 2 was open for 7 weeks.

### Stage 4: analysis for prioritisation

Before any analysis, scores were separated from responder characteristics. The group supported equal weighting of the two criteria and presenting of a single ranked ordering of the avenues, irrespective of research domain. The CHNRI method suggests summing mean scores for each criterion to calculate scores for ranking. Normality testing was done by CLLM who was provided with the data with anonymised identifiers rather than avenue labels to carry this analysis out in a blinded manner and avoid bias. This normality testing (Skewness, Kurtosis, Royston χ_2_, Shapiro-Wilk W, and Shapiro-Francia W'; Stats Direct version 3.3.5) of the raw score data showed that most criterion score responses did not follow a normal distribution. Therefore, the analysis plan was amended to calculate the median rather than the mean of the criterion scores, range and interquartile range were adopted as measures of dispersion, and box and whisker plots were used to show data dispersion.

For each research avenue, a median criterion score was calculated for each of the two criteria, and then the two median criterion scores summed to create a total score; ie, for each research avenue:

Median important knowledge score = median of all importance scores for that criterion

Median impact score = median of all impact scores for that criterion

Total score = median important knowledge score (k) + median impact score (i)

The total score was then used to present the research avenues in rank order from highest to lowest. Median scores for each avenue were calculated for responder groups to aid interpretation, but not for ranking. All available data from all respondents completing the survey, in full or in part, were analysed. Unsure or missing responses were not allocated a score and not included in the data from which the median was derived. Descriptive statistics were used to summarise the demographic data of participants, response rates for each criterion, and research avenues. To indicate dispersion of scores within the same rank, box and whisker plots were used with research avenues within rank, ordered alphabetically.

## Results

### Stage 1: gathering research uncertainties (e-survey 1)

The first survey received a total of 213 respondents (table 1). People with a musculoskeletal disorder were the highest responders, accounting for 76 respondents (36%). Clinical researchers (n=47 [22%]), health-care professionals (n=41 [19%]), and non-clinical researchers (32 [15%]) formed most of the remaining responses.

**Table 1:** Characteristics of responders to both e-surveys

	**E-survey 1 (n=213)**	**E-survey 2 (n=189 completed demographic data)**
**Gender**		
Male	NA	60 (32%)
Female	NA	117 (62%)
Unstated	NA	12 (6%)
**Ethnicity**		
White: British, English, Scottish, Welsh, or Northern Irish	NA	131 (69%)
Any other white background	NA	17 (9%)
White: Irish	NA	4 (2%)
Asian or Asian British: Indian	NA	2 (1%)
Any other Asian background	NA	2 (1%)
Black or Black British: African	NA	2 (1%)
Mixed (please state)	NA	2 (1%)
Asian or Asian British: Pakistani	NA	1 (1%)
Black or Black British: Caribbean	NA	1 (1%)
Asian or Asian British: Chinese	NA	1 (1%)
Any other ethnic group	NA	1 (1%)
Skipped the question	NA	25 (13%)
**Age, years**		
18–29	NA	5 (3%)
30–39	NA	21 (11%)
40–49	NA	37 (20%)
50–59	NA	50 (26%)
60–69	NA	40 (21%)
70–79	NA	25 (13%)
80–89	NA	4 (2%)
Skipped the question	NA	7 (4%)
**Nature of interest in musculoskeletal disorders**		
Person with a musculoskeletal disorder or patient	76 (36%)	64 (34%)
Member of public with an interest in a condition or area	6 (3%)	8 (4%)
Carer	0	2 (1%)
Patient support organisation	1 (<1%)	1 (1%)
Health-care professional	41 (19%)	43 (23%)
Clinical researcher	47 (22%)	29 (15%)
Non-clinical researcher	32 (15%)	29 (15%)
Charity or funding agency	0	1 (1%)
Industry or commercial	4 (2%)	0
Policy maker or government agency	0	0
Skipped the question	0	1 (1%)
Other (specified in free-text box)	6 (3%)	11 (6%)

NA=not applicable, question not included in survey.

Most participants entered uncertainties under each of the four domains (192 for diagnosis [90%], 200 for living well [94%], 203 for mechanisms [95%], and 184 for translation [86%]); additionally, 70 (33%) entered uncertainties in the ‘other’ uncategorised section.

Preference for research domain was ranked by 86% (184) of respondents. Understanding the causes and development of musculoskeletal disorders (mechanisms) was ranked as the most important overall by 57 respondents (31%). The other three categories had similar proportions of responders scoring as most important: 42 for diagnosis (23%), 42 for living well (23%), and 43 for translation (23%).

### Stage 2: consolidating uncertainties and generating research avenues

**Figure 2:**
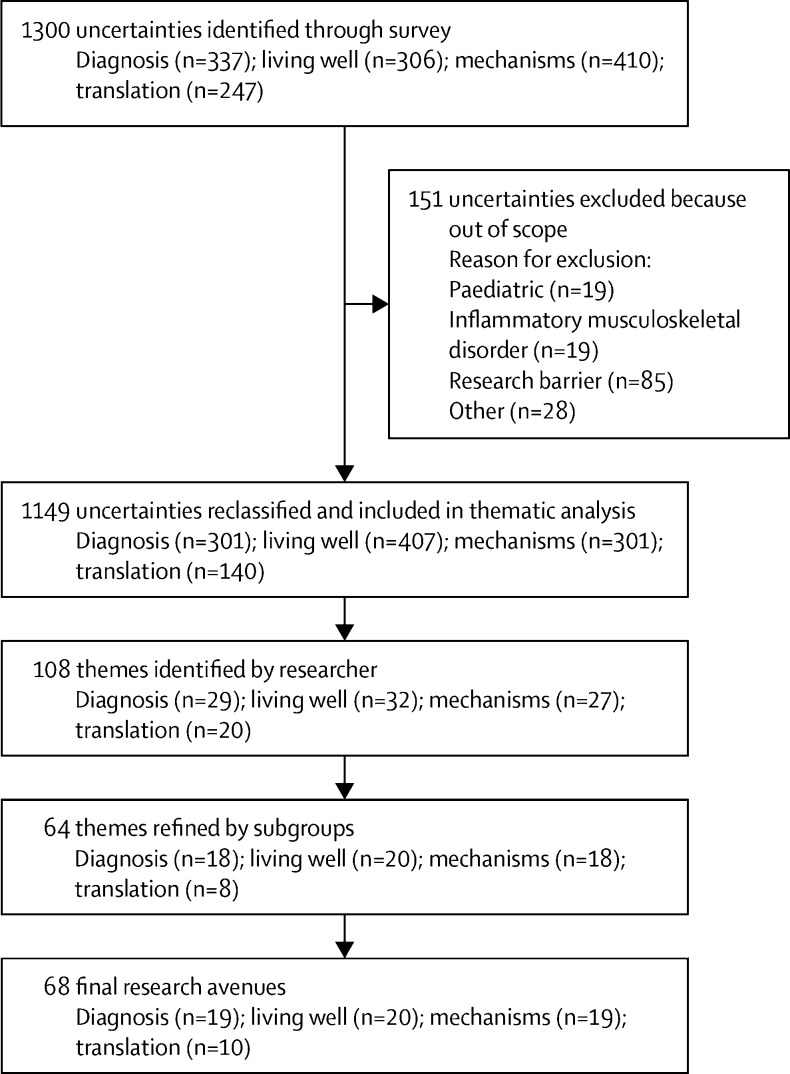
Stage 2—process of consolidation of uncertainties and generation of research avenues

Following separation of discrete research uncertainties and reclassifying the ‘other’ category into one of the four domains, a total of 1300 uncertainties (337 for diagnosis, 306 for living well, 410 for mechanisms, and 247 for translation) were submitted by the 213 respondents (mean 6·1 per respondent). Respondents entered 0–18 uncertainties per domain (mean per domain: 1·7 for diagnosis, 1·5 for living well, 2·0 for mechanisms, and 1·3 for translation). A total of 151 uncertainties were considered out-of-scope for the exercise, not answerable by research or highlighting barriers to research (to be explored separately in a future publication or charity report). The final number of research uncertainties was therefore 1149 (figure 2). At researcher review, 222 uncertainties were moved between domains, leading to: 301 for diagnosis, 407 for living well, 301 for mechanism, and 140 for translation.

Initial review consolidated these uncertainties into 108 draft themes (figure 2), which were further refined to 64 themes after subgroup review (appendix 2 pp 13–17). Of note, themes relating to personalised or stratified care, prognosis, prevention, health-care professional education, genetic influences, and delivering standardised care appeared in more than one domain. However, although the themes were duplicated, the focus of the uncertainties was different in each domain.

During the subgroup meetings and whole group workshop it became clear that most uncertainties and themes related to overarching issues that were relevant to many, if not all, musculoskeletal disorders. A decision was made to phrase all research avenues without reference to specific musculoskeletal disorders to be as generalisable and inclusive as possible. Similar questions were further consolidated, and questions where they occurred in different domains were separated, leading to a final list of 68 research avenues. Of these, 19 were in diagnosis, 20 in living well, 19 in mechanisms, and ten in translation (figure 2).

### Stage 3: scoring the research avenues (e-survey 2)

The second survey was live from Aug 16 to Oct 3, 2021. 114 respondents from e-survey 1 who opted to receive further contact about a second survey were invited to participate. A total of 285 people answered questions in e-survey 2. A further 197 people gave consent to take part but did not contribute any survey data.

Of those who contributed data, 189 (66%) (table 1) completed in full and a further 96 people (34%) partly completed the survey (more than one question completed, mean 36 responses, range 28–42).

The largest group of respondents represented lay people (patients, carers, and members of the public with an interest in the condition; n=74 [39%]), followed by researchers (n=58 [31%]) and health-care professionals (n=43 [23%]). The respondents were from a range of age groups and represented male and female sexes, although more females than males completed the survey (table 1). Information on ethnicity was not provided by a proportion of respondents (n=25 [13%]). Where this information was provided, 152 (93%) were White. Where respondents were researchers, a wide range of stages of research was represented (appendix 2 p 20). There was only one responder (1%) who reported representing charity or funding agencies, with no responders reporting representing industry industry such as pharmaceutical or medical technology companies.

The responses for partial respondents were quite evenly distributed across the questions: the mean number of responses per criterion question were 215 for diagnosis and Impact, 210 for living well, 217 for mechanisms, and 207 for translation. The minimum number of responses for a criterion question was 200 and the maximum number 227. Considering the completeness of data, of 30 964 answered questions there were 1296 (4·2%) unsure responses and 670 (2·2%) skipped responses (appendix 2 pp 21–35).

### Stage 4: analysis for prioritisation

A ranked list was produced from the complete responders plus any partial responders for each avenue, on the basis of the total scores for each research avenue (table 2). Considering a maximum possible score of 20 (10 + 10), and minimum score 2 (1 + 1), the highest-ranking research avenue was “Develop and test new treatments to prevent or reduce progression of musculoskeletal disorders” with a score of 18. The second ranking avenue with a score of 16·5 was “Identify the best ways to manage pain and/or improve quality of life”. The next 23 research avenues all tied in third ranking with a score of 16 and covered a range of areas. There was a total of eight different rankings, with scores ranging between 12 and 18. The minimum score was equivalent to a median score of 6/10 for each criterion, meaning that all research avenues had moderate to high scores (appendix 2 pp 36–38).

**Table 2:** Ranked research avenues

	**Score**	**Domain**
**Rank 1**		
Develop and test new treatments to prevent or reduce progression of musculoskeletal conditions.	18·0	LW
**Rank 2**		
Identify the best ways to manage pain or improve quality of life.	16·5	LW
**Rank 3**		
Develop and test approaches to help people with MSK conditions make lasting changes to improve their health.	16·0	LW
Develop and test ways to target and personalise treatments to each individual.	16·0	LW
Find out how to improve accurate and earlier diagnosis of MSK conditions.	16·0	D
Find out more about the benefits, safety, and best ways to use exercise or rehabilitation.	16·0	LW
Identify biological targets to develop new treatments that change the course of disease.	16·0	M
Identify disease processes that will allow better targeting of treatment to improve people's outcome.	16·0	M
Identify how to earlier predict the progress of MSK conditions.	16·0	M
Identify the best way to deliver the best support and information to help people effectively self-manage their condition.	16·0	LW
Identify the surgical techniques, technologies, and implants that help people the most.	16·0	LW
Identify tools, tests, and markers that can diagnose disease at an early stage and inform whether the disease will progress and respond to treatment.	16·0	M
Investigate how best to combine treatments.	16·0	LW
Study biological disease processes to identify ways to predict whether MSK conditions will develop or existing conditions will progress during someone's lifetime.	16·0	M
Study how chronic pain develops.	16·0	M
Study how new clinical, biological, and genetic and technology approaches can monitor the effectiveness of treatments.	16·0	T
Study how new clinical, biological, genetic, and technology approaches can improve diagnosis.	16·0	T
Study the effect of lifestyle (eg, work and exercise) on how MSK conditions develop and progress.	16·0	M
Study whether a better understanding of disease processes can be used to develop new ways of preventing MSK conditions.	16·0	M
Test new ways of making sure the right person gets the right treatment.	16·0	D
Understand disease processes so that we can better identify differences (subgroups of people) within the same condition.	16·0	M
Understand how MSK tissues repair themselves and how this could be enhanced to improve MSK conditions.	16·0	M
Understand the best ways of providing research-proven treatments to people, including where, when, and by whom.	16·0	T
Understand the links between tissue damage and pain.	16·0	M
Understand why people's pain experiences are different and why some people develop chronic pain when others do not.	16·0	M
**Rank 4**		
Understand the biological links between MSK conditions and other illnesses.	15·5	M
**Rank 5**		
Develop and test approaches to identify and help people with MSK conditions who need psychological support.	15·0	LW
Develop better ways to overcome the known difficulties in the process of turning a possible effective treatment into a safe, licensed product.	15·0	T
Find better ways to speed up the uptake of research results into treatment guidelines and policy.	15·0	T
Identify and find ways to address gaps in health-care professional knowledge about MSK conditions.	15·0	LW
Identify the best approaches to improving communication about MSK conditions between patients and their health-care professionals.	15·0	LW
Identify the best ways to improve outcomes after surgery.	15·0	LW
Improve how information from lab-based research and clinical trials is used to safely speed up making the best treatments available.	15·0	T
Investigate ways to speed up the process of turning scientific research findings into effective treatments.	15·0	T
Study how diet and gut bacteria can change the risk of developing MSK conditions.	15·0	M
Study how genes or ethnicity affect how MSK conditions develop.	15·0	M
Study how injury can lead to an increased risk of developing MSK conditions.	15·0	M
Study the role of inflammation in non-inflammatory MSK conditions.	15·0	M
Understand and overcome the barriers preventing research-proven tests and treatments being put into practice.	15·0	T
Understand how key biological disease processes drive development and progress of MSK conditions.	15·0	M
Understand how sex hormones and menopause change the risk of MSK conditions.	15·0	M
**Rank 6**		
Better understand the benefit, safety, and use of existing medicines, including injections.	14·0	LW
Decide the best ways of delivering remote care for people.	14·0	LW
Define the risk factors in MSK conditions that might predict important outcomes or enable screening.	14·0	D
Identify any groups of patients or patterns within a condition that inform on the course or outcomes of MSK conditions.	14·0	D
Identify the best aids, supports, and other devices to help people live well.	14·0	LW
Identify the best lifestyle interventions.	14·0	LW
Set up and test new ways of using electronic health records for accurate, earlier diagnosis, and personalised monitoring.	14·0	D
Set up and test whether patients holding their own health data records helps them better manage their condition and make decisions with their clinician.	14·0	D
Study whether making changes to risk factors can prevent or delay the start of MSK conditions.	14·0	D
Study the best way of bringing together scientists, clinicians, industry, policy makers, and people with MSK conditions to improve the development of early research towards better available treatments.	14·0	T
Study the best way of sharing the results of research with clinicians, scientists, policy makers, and people with MSK conditions.	14·0	T
Study the best ways to measure the true effects of MSK conditions on individuals.	14·0	D
Test artificial intelligence approaches to analyse electronic health records and other large databases, (eg, of x-rays or scans) to improve care.	14·0	D
Test how clinical tools, tests, and markers can improve diagnosis.	14·0	D
Understand and address the reasons why everyone with a particular MSK condition does not have the same access to care.	14·0	LW
Understand and address the reasons why everyone with MSK conditions does not receive minimum standards of care.	14·0	LW
Understand and address the reasons why people have difficulty or delays accessing care.	14·0	LW
Understand and meet people's needs for monitoring and review of their condition.	14·0	LW
Understand how having an early diagnosis affects people, health care, and society.	14·0	D
Understand the best ways to diagnose and describe MSK conditions. Define features of relevant smaller groups within the same condition which make a difference to outcomes or care.	14·0	D
Understand the links between MSK and other long-term conditions, and the effect they have on people, work, and society.	14·0	D
Understand the reasons why diagnosis is sometimes delayed, and how best to reduce delays.	14·0	D
**Rank 7**		
Find out how to produce a better estimate of the true cost of long-term MSK conditions to people and society.	13·0	D
Understand how changes in society, work, and people's circumstances, including finances, might lower the risk or effects of MSK conditions.	13·0	D
**Rank 8**		
Study the pros and cons of screening for MSK conditions for people, health care, and society.	12·0	D
Study whether increasing public awareness of MSK conditions will encourage people to have a healthier lifestyle, get an earlier diagnosis, and better care.	12·0	D
Study whether new ways of collecting and using standard health data will improve people's care and help society.	12·0	D

Within each rank, avenues are presented in alphabetical order. LW=living well. D=diagnosis. T=translation. M=mechanisms.

The top three rankings (scores 16 and higher) comprised of 25 research avenues, which included a mixture of all four research domains, with a preponderance of questions from mechanisms (n=12) and from living well (n=8). Furthermore, no research avenues from mechanisms were included within ranks 6–8. When examining the median scores by responder characteristics, there was little difference between lay, clinical, and researcher responders for the top-ranking research avenues (appendix 2 pp 21–35). However, differences between researcher and lay respondents' median scores were larger (3 or 4 points) in avenues concerning research in overcoming barriers to the implementation of care, or access to care, which researchers tended to score as less important.

## Discussion

If research is to address and resolve important questions and lead to impact, the identification and development of research questions must involve those people affected by the research.^30^ This exercise set out to include all relevant stakeholders in a valid, equitable, transparent, and predefined process to define, score, and then rank research priorities across all stages of research in a wide range of musculoskeletal disorders.

The process identified the top priority as developing new treatments that prevent or reduce progression of these diseases. This outcome is important as, conversely, much recent research and clinical management focus has been on optimising existing treatments, self-management, or strategies to manage symptoms. The association of common musculoskeletal disorders with ageing contributes to the normalisation, tolerance, and de-prioritisation of these disorders among individuals.^38^, ^39^ These views possibly permeate across government, health-care systems, and society, leading to an acceptance of the occurrence of musculoskeletal disorders, the associated pain, loss of function, and relative paucity of treatment options. The top ranking of this research avenue sends a clear message that our respondents felt efforts must be increased to identify novel approaches to treat underlying disease process and to reject this status quo.

We also identified key themes relating to better understanding and management of pain; improving disease prevention, accurate diagnosis, and prediction; and understanding how to better implement, personalise, target, combine, and monitor treatments, with a key emphasis on understanding the underpinning mechanisms. Musculoskeletal pain has also been highlighted as important via insight gathering by Versus Arthritis, and stratified medicine by UK Research Council funding calls.^40^ Despite the relative paucity of discovery science questions in previous priority setting exercises,^32^ and the assumption that lay responders are less likely to prioritise discovery science questions that might be further from patient benefit than applied research,^41^ research about disease mechanisms was rated as the most important domain in e-survey 1, and mechanistic research avenues dominated the top three ranks. Of note, we also included priorities relating to health-care services, implementation, and economic factors with overcoming barriers to implementation of evidence-based treatments ranking highly (rank 3); these areas have been noted to be underrepresented in previous musculoskeletal priority setting exercises, although the need for implementation studies is increasingly recognised.^32^, ^42^ We identified that, in general, lay responders rated avenues higher than researchers. However, researchers tended to particularly rate ‘other’ avenues concerning overcoming barriers to implementation of, or access to, care of lower importance than lay responders. The reasons for this difference in rating are unclear, but it is possible that academic responders are less aware of, or do not sufficiently value, health services and implementation research.^43^

We would not encourage comparison of the performance of the research domains as there was substantial overlap between them and we adopted a flexible approach to moving uncertainties between domains. As an example, the top-ranking research avenue relating to developing and testing new treatments was identified within living well but was highly relevant to translation.

To our knowledge, this exercise is the first of its kind to identify and rank priorities for research across musculoskeletal disorders, from discovery science to applied clinical and health research, including translation. Further strengths of this process were that it was robust, transparent, disease and research stage agnostic, and included a wide range of stakeholders at all stages. The whole process was overseen by an expert steering group with a final ranking process that was independent of them. Public contributors played a vital role throughout, particularly to optimise accessibility and minimise the length of the second survey.

There were some limitations to this exercise. We chose not to produce a top ten or top 20 priorities list, which might be more easily disseminated; we chose not to select a threshold above which to highlight top scores, as all 68 of the research avenues received high to moderate scores. This result is perhaps not surprising, as those with the lowest ranking were still derived from themes arising from multiple respondents' priorities in e-survey 1. The separation between the highest and lowest score was relatively modest (a difference of 6 points out of a possible 18—ie, maximum possible total score of 20, minus minimum possible total score of 2). The seemingly large number of possible research avenues appears justifiable, given the wide range of disorders and research stages, and the high prevalence of unmet needs. Arguably, our predefined research domains might not represent the full remit of musculoskeletal research and could have biased responses to e-survey 1; however, we felt that these domains adequately covered the breadth of all uncertainties elicited, and areas submitted under the ‘other’ category could be assigned to these four domains. Although this priority setting exercise has addressed some limitations previously identified in a scoping review of priority setting with regards to research topics for musculoskeletal conditions,^32^ economic evaluation was perhaps underrepresented and might need to be considered as the research avenues are further refined. Due to the COVID-19 pandemic, the entire exercise was done electronically as it was not feasible at the time to collect data by other means—eg, in person or paper-based questionnaires, meaning that some people might have been excluded from taking part. Although we reached a good balance in terms of accessing our target groups and many lay respondents, there appeared to be a bias towards women, White respondents, and people living in England (as opposed to the devolved nations of the UK), and with a paucity of respondents from industry or pharmaceutical companies, or representing carers, suggesting the processes put in place for advertising did not sufficiently reach these groups. In addition, the lack of demographic data (none from partial responders) limits our ability to make conclusions about the generalisability of the sample. Finally, we made some important intentional departures from the CHNRI method. These included using only two criteria with which to score, to minimise burden of the survey and ensure all criteria were understandable to all stakeholders. In addition, our scoring methods needed to be refined, following the publication of the protocol, to use median criterion scores rather than mean scores. Our exercise was the first instance that we could find within a CHNRI-type process where the distribution of data and appropriateness of use of a mean as a component of a summary score had been scrutinised, showing the use of means were not appropriate for these data. Mean values are included within the box and whisker plots showing that on some occasions the mean score, which is affected by skewness of data, would have influenced rank. The distribution of data might have been influenced by our decision to use a numeric rating scale rather than yes or no responses; nonetheless, our experience suggests other researchers using the CHNRI method need to consider data distribution in their plans for analysis. Furthermore, as rating scales were ordinal, parametric analysis was not appropriate.

This prioritisation exercise provides a source of information and evidence but should also be a call to action for funders to support research priorities in musculoskeletal disorders research, and for researchers and health-care professionals to consider stakeholder views on what might be important and impactful. As these avenues are developed into specific research questions or projects, it is vital that stakeholders, including public contributors, are involved at all stages in this process, and that these priorities are reviewed to identify the extent to which they have informed subsequent relevant funding calls.^32^ There is also a need to address the many barriers and challenges to successful research in this area. There is undoubtedly some overlap between barriers and some of the priorities in the translation domain: regulatory or policy changes might be needed to address these.^42^

In summary, we have presented the prioritisation process and associated scores of 68 identified research avenues in ranked order of their combined likelihood of leading to important new knowledge and impact. These research uncertainties and their rankings were generated by a mixed stakeholder user group, including public contributors, health practitioners, and researchers. Further work is now needed to translate these priorities into researchable questions for calls by funders, in the UK and elsewhere. We will seek to disseminate and develop these priorities and audit their uptake. Specifically, the Musculoskeletal Research Advisory Group and Versus Arthritis are committed to further develop the top priorities, alongside funders and stakeholders, into more specific questions within avenues, or translation of the avenues into population, intervention, comparison, and outcome questions where possible. In the meantime, we hope the findings and key themes we have summarised will empower the research community (including public contributors) to identify what is important within their own fields of work. We expect this work to catalyse greater attention and funding of high-quality research leading to improved knowledge and impact in the understanding and management of musculoskeletal disorders.

## Declaration of interests

FEW reports grants from UK Research and Innovation (UKRI), National Institute for Health and Care Research (NIHR), Versus Arthritis, Medical Research Council (MRC); consulting fees from Pfizer; and committee role for MRC and NIHR. ZP reports grants from NIHR, Versus Arthritis, Royal Osteoporosis Society, General Council for Nursing, Haywood Foundation, Chartered Society for Physiotherapy, and British Society for Rheumatology; honoraria from the Royal College of General Practitioners; non-paid consultancy for UCB, and unpaid committee roles with Royal Osteoporosis Society and Haywood Foundation. CB reports grants from Versus Arthritis and University of Liverpool (Liverpool, UK). EMC reports grants from Royal Osteoporosis Society, Elizabeth Blackwell Institute (University of Bristol, Bristol, UK) MRC Confidence in Concept Award, NIHR, Southmead Hospital Charity Research Fund, Healthcare Quality Improvement Partnership; payment or honoraria from Danske Fysioterapeuter and Journal of Bone and Mineral Research Plus; and an unpaid committee role with the Royal Osteoporosis Society. MG reports grants from Versus Arthritis and Clarendon Fund. RKJ reports grants from the Ministry of Defence. CLLM reports grants from H2020 ITN, Disc4All, Flexion Plc (Contract Research Funding), Engineering and Physical Sciences Research Council (EPSRC), MRC, and Versus Arthritis; consultancy fees and royalties or licensing fees from Flexion; committee roles for *JOR Spine*, Discovering Innovative Solutions for Conditions of the Spine, and Society of Back Pain Research. JL reports grants from MRC, The John George William Patterson Foundation, and the Ruth & Lionel Jacobson Charitable Trust. DJM reports grants from Orphelion, Innovation for All, Welsh Government with Knoell, Versus Arthritis, KESS2 & Hospital Innovations, EPSRC, Wellcome Trust Collaborative Award, Petplan Charitable Trust, and British Veterinary Orthopaedic Association; payment or honorarium from Eli Lilly; receipt of drugs from Novartis; participation on a data safety oversight board for NIHR (paid) and Innovative Medicines Initiative (paid), unpaid leadership role within British Orthopaedic Research Society and Orthopaedics Research Society, and paid committee role in Science Foundation Ireland Early Career Panel. NLM reports grants from MRC and Chan Zuckerberg Initiative. HP reports grants from Knowledge Transfer Partnership–Innovate UK, Pacira Pharmaceuticals, Zimmer Biomet Healthcare, B Braun, and Wellcome Trust; consulting fees from Medacta International, Smith and Nephew, Depuy Synthes, JRI Orthopaedics, Janssen, Meril Life, Zimmer Biomet, and Paradigm Pharmaceuticals; payment or honoraria from Invibio; payment for expert testimony from Kennedy's Law; support for attending meetings from Medacta International; participation on data safety oversight board for GreenBric Study; unpaid committee role for NIHR and National Joint Registry Regional Clinical Coordinators; and receipt of drug from Pacira Pharmaceuticals. GP reports grants from NIHR, Nuffield Foundation, and Versus Arthritis, and consulting fees from Office Health Inequalities & Disparities; participation on data safety monitoring board and role as *Lancet* Osteoarthritis Commissioner. SMR reports grants from Versus Arthritis and Wellcome Trust, honoraria from Portugal grant review, and had a role as treasurer and committee member for the Tissue and Cell Engineering Society. EJS reports grants from NHSX and committee role on musculoskeletal reform. LT reports grants from Versus Arthritis, The Macular Society, MRC, Rosetrees Trust, University of East Anglia Faculty of Medicine and Health Sciences (Norwich, UK), and the Kennedy Institute of Rheumatology; support for attending meetings from Advanced Course–Matrix Pathobiology, Signalling and Molecular Targets, and OACON 2019. RKW reports grants from EPSRC, DePuy Synthes, and Versus Arthritis; unpaid committee role in Royal Academy of Engineering; and receipt of materials from DePuy synthesis. CW reports support for attending meetings from European Pain Federation; participation on advisory board for University of Swansea (Swansea, UK), Health Data Research UK, and National Institute for Health and Care Excellence; and committee role for Versus Arthritis and University of Bath (Bath, UK). CF is employed by Versus Arthritis. All other authors declare no competing interests.
