# Supplementary material for: Research priorities to reduce the impact of musculoskeletal disorders: a priority setting exercise with the child health and nutrition research initiative method
Source: Lancet Rheumatol. 2022 Jul 27;4(9):e635–45. doi: 10.1016/S2665-9913(22)00136-9 (PMC9584828; doi:10.1016/S2665-9913(22)00136-9)
Supplement: Supplementary appendix 1 [file mmc1.pdf]

THE LANCET
Rheumatology

## Supplementary appendix 1

This appendix formed part of the original submission and has been peer reviewed. We post it as supplied by the authors.

Supplement to: Paskins Z, Farmer CE, Manning F, et al. Research priorities to reduce the impact of musculoskeletal disorders: a priority setting exercise with the child health and nutrition research initiative method. *Lancet Rheumatol* 2022; published online July 26. https://doi.org/10.1016/S2665-9913(22)00136-9.

| AREA OF INSIGHT | NAME | ROLE | AFFILIATION |
|---|---|---|---|
| Academic Clinician- Osteoporosis and Bone | Dr Zoe Paskins | Reader, Honorary Consultant Rheumatologist | Keele University |
| People with arthritis | Colin Wilkinson | Patient Representative | Versus Arthritis - Research Patient Representative Group |
| People with arthritis | Amanda Clark | Patient Representative | Versus Arthritis - Research Patient Representative Group |
| People with arthritis | Debra Dulake | Patient Representative | Versus Arthritis - Research Patient Representative Group |
| People with arthritis | Jane Taylor | Patient Representative | Versus Arthritis – Research Patient Representative Group |
| Non clincial Researcher - Epidemiologist | Professor George Peat | Professor of Clinical Epidemiology | Keele University |
| Non clincial Researcher - Biomechanics | Professor Richard Jones | Professor of Clinical Biomechanics | University of Salford |
| Non clincial Researcher - Biomedical Engineering | Professor Ruth Wilcox | Professor of Biomedical Engineering | University of Leeds |
| Non clincial Researcher - Regenerative Medicine | Dr Stephen Richardson | Senior Lecturer, Division of Cell Matrix Biology & Regenerative Medicine | University of Manchester |
| Non clincial Researcher - Cell Biology | Professor Christine Le Maitre | Professor of Cell Biology and Tissue Regeneration | Sheffield Hallam University |
| Non clincial Researcher - Genetics (DEPUTY) | Professor John Loughlin | Professor of Musculoskeletal Research | Newcastle University |
| Non clincial Researcher - Molecular pathogenogenesis | Dr Linda Troeberg | Senior Lecturer | University of East Anglia |
| Non clincial Researcher - Neuroscience, pain | Dr David Andersson | Senior Lecturer | King's College London |
| Non clinical Researcher - Psychology | Dr Felicity Bishop | Associate Professor, Psychology | University of Southampton |
| Translational Scientist - Signalling mechanisms | Professor Debbie Mason | Reader, School of Biosciences; Director of Pre-Clinical Research | Cardiff University |
| Academic Clinician – Surgeon | Professor Hemant Pandit | Director of Research and Innovation | University of Leeds |
| Academic Clinician – Surgeon | Professor Neal Millar | Clinical Senior Lecturer (Immunology) | University of Glasgow |
| Academic Clinician – Rheumatologist, Osteoporosis | Professor Emma Clark | Professor of Musculoskeletal Epidemiology | University of Bristol |
| Academic Clinician – Rheumatologist, Osteoarthritis (LEAD) | Dr Fiona Watt | Reader in Rheumatology, Honorary Consultant Rheumatologist | Imperial College London |
| Academic Clinician - Allied Health Professional | Dr Emma Salt | Consultant Physiotherapist | University Hospitals of Derby and Burton NHS Foundation Trust |
| Academic Clinician - Psychological sciences | Dr Christopher Brown | Senior Lecturer, Psychological Sciences | University of Liverpool |
| Clinician - GP | Dr Elspeth Wise | General Practitioner | Talbot Medical Centre, South Shields (Newcastle) |
| Clinician - Rheumatologist | Dr Maura McCarron | Rheumatologist | Belfast Health and Social Care Trust |
| Trainee Clinician - Surgeon | Dr Tim Barlow | Trainee orthopaedic surgeon | University Hospitals Coventry and Warwickshire NHS Trust |
| Trainee Clinician - Rheumatologist | Dr Malvika Gulati | D Phil student | University of Oxford |
