# Supplementary material for: Research priorities to reduce the impact of musculoskeletal disorders: a priority setting exercise with the child health and nutrition research initiative method
Source: Lancet Rheumatol. 2022 Jul 27;4(9):e635–45. doi: 10.1016/S2665-9913(22)00136-9 (PMC9584828; doi:10.1016/S2665-9913(22)00136-9)

THE LANCET
Rheumatology

Supplementary appendix 2

This appendix formed part of the original submission and has been peer reviewed. We post it as supplied by the authors.

Supplement to: Paskins Z, Farmer CE, Manning F, et al. Research priorities to reduce the impact of musculoskeletal disorders: a priority setting exercise with the child health and nutrition research initiative method. *Lancet Rheumatol* 2022; published online July 26. https://doi.org/10.1016/S2665-9913(22)00136-9.

Research priorities to reduce the impact of Musculoskeletal Disorders

Supplementary Data File

Contents

1. Text for E-Survey 1 2

2. Text for E-Survey 2 5

3. Recruitment routes 12

4. Themes from researcher review 13

   Diagnosis 13

   Living Well 14

   Mechanisms 15

   Translation 16

      Early Translation 16

      Late Translation 16

5. Themes from subgroup review 17

   Diagnosis 17

   Living well 18

   Mechanisms 19

   Translation 19

6. Researcher breakdown results from E-survey 2 20

7. Data completeness and Median scores by responder characteristics 21

8. Median, means and distributions of each of the criterion scores for each research avenue 36

1. Text for E-Survey 1

MSKRAG_SurveyText_14Aug2020

Mechanisms in musculoskeletal disorders research

This covers areas including the following:

- Understanding the causes and development of musculoskeletal disorders
- Disease processes shared between disorders

In your opinion, what are the important unanswered questions or uncertainties that could be answered by research in this area?

2. Diagnosis and impact in musculoskeletal disorders research

This covers areas including the following:

- Achieving an early and accurate diagnosis
- Measuring the true impact of musculoskeletal disorders on individuals and on society
- Maximising the potential of electronic health records

In your opinion, what are the important unanswered questions or uncertainties that could be answered by research in this area?

3. Managing and living well with musculoskeletal disorders

This covers areas including the following:

- Improving self-management and support in the home or community
- Improving any aspect of healthcare treatment

In your opinion, what are the important unanswered questions or uncertainties that could be answered by research in this area?

4. Successfully turning research findings into innovations that help people living with musculoskeletal disorders

This covers areas including the following:

- Ensuring that research-proven tests, treatments and approaches are routinely available in clinical practice
- Enabling discoveries to move from the laboratory to the clinic, towards patient benefit

In your opinion, what are the important unanswered questions or uncertainties that could be answered by research in this area?

5. In your opinion, what are the challenges or barriers to progress in these four areas which could be overcome?

6. Are there any other important questions or challenges that you wish to highlight?

We would like to gather some information on your background to ensure balance and diversity in this exercise. We also hope to understand whether you would like to be involved in any further activities.

7. Considering the areas of musculoskeletal disorders research we asked you about, please rank them in the order of importance to you:

- Understanding the causes and development of musculoskeletal disorders
- Diagnosis and impact of musculoskeletal disorders
- Managing and living well with musculoskeletal disorders
- Successfully turning research findings into tests and treatments that help people living with musculoskeletal disorders
- Other areas that I have highlighted

8. Are you happy to be contacted to consider participating further in the following activities?

    - Follow-up online rating of research themes from this exercise (this activity could take up to one hour)
    - Small group activity online or by phone
    - Large group activity/webinar online
    - Discussion by email
    - Any of above
    - None of the above
    - I am uncertain at this time

If you are happy to be contacted about this, please give your email here (this will only be used to contact you about the activity you selected above):

9. I am a (tick the one that most applies):

    - researcher (clinician)
    - researcher (non clinician)
    - healthcare professional
    - charity/funding agency
    - person with a musculoskeletal disorder/patient
    - member of public with an interest in a condition or area
    - patient support organisation
    - carer
    - industry/commercial
    - policymaker/government agency
    - other
    - If other, please give details:

10. Who is your main employer?

    - NHS
    - university
    - industry
    - charity/funder
    - government
    - other
    - not applicable/prefer not to say
    - If other, please give details

11. What is your Institution/organisation/place of employment (answering this question will help us check for geographical representation) (optional)

12. If you are a researcher, what type of research do you carry out (select all that apply):

- fundamental/mechanistic/laboratory
- translational (at any stage)
- clinical
- epidemiological/statistical/big data
- implementation science
- health services research
- qualitative research
- other
- not applicable
- If other, please give details

13. Please give up to four key words that describe your MSK disorder interests and/or research area (optional)

Thank you for completing our research prioritisation exercise. Your contributions will help us to shape the top priorities for future research into musculoskeletal disorders.
Please contact us if you have any queries <insert email address>.
The Musculoskeletal Disorders Research Advisory Group Versus Arthritis

<link to share survey on social media>

2. Text for E-Survey 2

MSKRAG_Survey2Text_V 2.0_11 August 2021

All areas apply specifically to people with musculoskeletal (MSK) conditions. Where we use the term 'people' or 'patient' we mean people or patients with MSK conditions. Where we say 'conditions' we mean MSK conditions.

It's ok to score from your own knowledge and perspective. If you feel unsure, please tick 'unsure' instead of giving a rating. The overall score for a statement will not be affected if you choose unsure.

**Criterion**	**Question**	**Explanation**
	These statements will be in column headers on each page of the survey.	These notes will be listed at the top of each page of the survey.
Importance	Will research in this area have potential to lead to important new knowledge?<br>(Where 1 is not likely and 10 is extremely likely)	It might help to consider<br>• Will this lead to significant new important knowledge or understanding, **at any stage of research?**<br>• What is already known or being studied in this research area?
Impact	Might research in this area make a difference?<br><br>(Where 1 is not likely and 10 is extremely likely)	It might help to consider<br>• Could this research lead to new or more effective treatment or tests?<br>• How much could this research result in patient benefit, whether in the short or long term?<br>• Consider the size of this impact, (e.g. you might score a small benefit for a large number of people, or a large benefit for a smaller number of people)?

This list of research areas is then presented in the survey for scoring (for each of the two criteria) with 2 'reminder' stems for the criteria:

- Will this research lead to important new knowledge?
- Will this research make a difference and lead to impact?

NB Questions will not be numbered in survey, as they will be presented in a random order within each of 4 research themes (from Survey 1).

Research Areas, page 1

1. Test artificial intelligence approaches to analyse electronic health records and other large databases, (e.g. of x-rays or scans) to improve care.

	2. Study whether new ways of collecting and using standard health data will improve people's care and help society.
	3. Set up and test whether patients holding their own health data records helps them better manage their condition and make decisions with their clinician.
	4. Set up and test new ways of using electronic health records for accurate, earlier diagnosis and personalised monitoring.
	5. Find out how to improve accurate and earlier diagnosis of MSK conditions.
	6. Understand how having an early diagnosis affects people, healthcare, and society.
	7. Understand the best ways to diagnose and describe MSK conditions. Define features of relevant smaller groups within the same condition which make a difference to outcomes or care.
	8. Test how clinical tools, tests and markers can improve diagnosis.
	9. Understand the reasons why diagnosis is sometimes delayed, and how best to reduce delays.
	10. Test new ways of making sure the right person gets the right treatment.
	11. Understand the links between MSK and other long-term conditions, and the effect they have on people, work and society.
	12. Study the best ways to measure the true effects of MSK conditions on individuals.
	13. Find out how to produce a better estimate of the true cost of long-term MSK conditions to people and society.
	14. Identify any groups of patients or patterns within a condition which inform on the course or outcomes of MSK conditions.
	15. Study if making changes to risk factors can prevent or delay the start of MSK conditions.
	16. Define the risk factors in MSK conditions that might predict important outcomes or enable screening.
	17. Study the pros and cons of screening for MSK conditions for people, healthcare and society.

18. Study whether increasing public awareness of MSK conditions will encourage people to have a healthier lifestyle, get an earlier diagnosis and better care.
19. Understand how changes in society, work and people's circumstances, including finances, might lower the risk or effects of MSK conditions.
Research Areas, page 2
20. Understand the links between tissue damage and pain.
21. Study how chronic pain develops.
22. Understand why people's pain experiences are different and why some people develop chronic pain when others do not.
23. Study how genes or ethnicity affect how MSK conditions develop.
24. Understand how sex hormones and menopause change the risk of MSK conditions.
25. Study biological disease processes to identify ways to predict if MSK conditions will develop or if existing conditions will progress during someone's lifetime.
26. Study how injury can lead to an increased risk of developing MSK conditions.
27. Study how diet and gut bacteria can change the risk of developing MSK conditions.
28. Study the effect of lifestyle (e.g. work and exercise) on how MSK conditions develop and progress.
29. Identify how to earlier predict the progress of MSK conditions.
30. Understand disease processes so that we can better identify differences (subgroups of people) within the same condition.
31. Identify disease processes that will allow better targeting of treatment to improve people's outcome.
32. Identify tools, tests and markers that can diagnose disease at an early stage and inform whether the disease will progress and respond to treatment.
33. Understand how key biological disease processes drive development and progress of MSK conditions.

34. Study the role of inflammation in 'non-inflammatory' MSK conditions.
35. Understand the biological links between MSK conditions and other illnesses.
36. Identify biological targets to develop new treatments which change the course of disease.
37. Understand how MSK tissues repair themselves and how this could be enhanced to improve MSK conditions.
38. Study whether a better understanding of disease processes can be used to develop new ways of preventing MSK conditions.
Research Areas, page 3
39. Understand and address the reasons why people have difficulty or delays accessing care.
40. Decide the best ways of delivering remote care for people.
41. Understand and address the reasons why everyone with MSK conditions does not receive minimum standards of care.
42. Understand and address the reasons why everyone with a particular MSK condition does not have the same access to care.
43. Develop and test ways to target and personalise treatments to each individual.
44. Identify the best way to deliver the best support and information to help people effectively self-manage their condition.
45. Identify and find ways to address gaps in healthcare professional knowledge about MSK conditions.
46. Identify the best approaches to improving communication about MSK conditions between patients and their healthcare professionals.
47. Develop and test new treatments to prevent or reduce progression of MSK conditions.
48. Investigate how best to combine treatments.
49. Better understand the benefit, safety and use of existing medicines, including injections.
50. Identify the best lifestyle interventions.
51. Identify the best aids, supports and other devices to help people live well.
52. Identify the surgical techniques, technologies and implants that help people the most.
53. Find out more about the benefits, safety and best ways to use exercise and/or rehabilitation.
54. Develop and test approaches to identify and help people with MSK conditions who need psychological support.
55. identify the best ways to improve outcomes after surgery.
56. Develop and test approaches to help people with MSK conditions make lasting changes to improve their health.

57. Understand and meet people’s needs for monitoring and review of their condition.
58. Identify the best ways to manage pain and/or improve quality of life.
Research Areas, page 4
59. Understand and overcome the barriers preventing research-proven tests and treatments being put into practice.
60. Understand the best ways of providing research-proven treatments to people, including where, when and by whom.
61. Study the best way of sharing the results of research with clinicians, scientists, policy makers and people with MSK conditions.
62. Find better ways to speed up the uptake of research results into treatment guidelines and policy.
63. Investigate ways to speed up the process of turning scientific research findings into effective treatments.
64. Develop better ways to overcome the known difficulties in the process of turning a possible effective treatment into a safe, licensed product.
65. Study the best way of bringing together scientists, clinicians, industry, policy makers and people with MSK conditions to improve the development of early research towards better available treatments.
66. Study how new clinical, biological, genetic and technology approaches can improve diagnosis.
67. Study how new clinical, biological and genetic and technology approaches can monitor the effectiveness of treatments.
68. Improve how information from lab-based research and clinical trials is used to safely speed up making the best treatments available.

We would like to gather some information on your background to ensure balance and diversity in this exercise.

1. I am a (tick the one that most applies):
    - researcher (clinician)
    - researcher (non clinician)
    - healthcare professional
    - charity/funding agency
    - person with a musculoskeletal disorder/patient
    - member of public with an interest in a condition or area
    - patient support organisation
    - carer
    - industry/commercial
    - policymaker/government agency
    - other
    - If other, please give details:

2. Who is your main employer?
    - NHS
    - university
    - industry
    - charity/funder
    - government
    - other
    - not applicable/prefer not to say
    - If other, please give details

3. Where is your Institution/organisation/place of employment (answering this question will help us check for geographical representation) (optional)

4. If you are a researcher, what type of research do you carry out (select all that apply):

    - fundamental/mechanistic/laboratory
    - translational (at any stage)
    - clinical
    - epidemiological/statistical/big data
    - implementation science
    - health services research
    - qualitative research
    - other
    - not applicable
    - If other, please give details

5. Please give up to four key words that describe your MSK disorder interests and/or research area (optional)

6. What is your age (optional)?

18-29
30-39
40-49
50-59
60-69
70-79
80-89
90 or over

7. What gender do you identify as (optional)?

Female
Male
Prefer not to answer

8. What is your ethnic group or background (optional)?

Thank you for completing Part 2 of our research prioritisation exercise. Your contributions will help us to shape the top priorities for future research into musculoskeletal conditions.
Please contact us if you have any queries <insert email address>
The Musculoskeletal Disorders Research Advisory Group Versus Arthritis
<link to share survey on social media

3. Recruitment routes

Route	Audience
Versus Arthritis website	General
Social Media (Versus Arthritis and University of Oxford	General
Versus Arthritis Research Newsletter	Research community (~800 people)
Versus Arthritis Research Partner Newsletter (lay audience)	Community of patients who work with Versus Arthritis (~100 people)
Versus Arthritis Network news	Healthcare Professionals (~9000 people)
Individual contacts of group members or Versus Arthritis	Varied – professional bodies, industry, policy makers, researchers, clinicians, patient groups.<br>To include:<br>Orthopaedic Research Society<br>British Orthopaedic Association<br>Fragility Fracture Network<br>OARSI<br>Society of Matrix Biology<br>OATECH<br>British Orthopaedic Research Society<br>APPROACH<br>Bone Research Society<br>European Orthopaedic Research Society<br>Royal Osteoporosis Society
Direct emails to those who gave permission to be recontacted (Survey 2 only)	Survey 1 respondents (114 people)

4. Themes from researcher review

Themes from researcher coding 18.2.21

Nb codes/themes in red appear across domains

Diagnosis

- Opportunities for EHR/big data -
    1. Machine learning/AI
    2. New approaches to collecting and organising routine data
    3. Patient held EHR and Linking to personal Data (phones)
    4. Using EHR for EPI/Research
    5. Using EHR to improve care - general
    6. Using EHR for prediction/personalised care
- EHR quality -
    7. Better EHR linkage
    8. Understanding Barriers to Quality (e.g. Coding)
    9. Improving quality
- Early Diagnosis -
    10. General comments
    11. Specific conditions
    12. Screening
    13. Prediction models
- Improving diagnosis -
    14. Refining definitions/diagnoses
    15. Tools/tests to support diagnosis (includes combination approaches)
    16. Specific imaging Qs
    17. Specific Biomarkers
- Barriers to diagnosis and referral -
    18. Understanding delays
    19. Overcoming barriers
    20. Diagnostic pathways
    21. Communication
- Personalised/stratified care – *also in mechanisms*
    22. Impact
    23. General/societal
    24. Individual
    25. Cost
- Prognosis – *also in mechanisms*
    26. Prognosis
- Prevention – *also in mechanisms*
    27. Prevention
- Public Health
    28. Public Health
- Other
    29. Other

Living Well

- Healthcare provision -
    1. Improving access
    2. Standardised care – also in translation
    3. Addressing inequalities
    4. Optimal care pathways – similar to models of care in translation
    5. Remote healthcare delivery
    6. Community and 3rd sector
    7. Monitoring and outcomes
- Approaches to care -
    8. Patient centred and holistic care
    9. Stratified care approaches
- Self-management -
    10. Supporting self-management (networks as well)
    11. Information
    12. Technology to support
    13. Nutrition
- Information -
    14. Access
    15. Provision
- Unmet treatment needs -
    16. General
    17. Osteoporosis and OI
    18. OA
    19. pain
- Surgery outcomes -
    20. Surgery outcomes
- Non-surgical Treatment -
    21. Complimentary treatment
    22. Combined treatments
    23. Drug safety/side effects
    24. aids and devices
    25. injections
    26. exercise (effective and safe)
    27. Psychological support
- Supporting behaviour change and adherence -
    28. Supporting behaviour change and adherence
- Predictors of treatment response -
    29. Predictors of treatment response
- HCP Education – *also in translation*
    30. HCP Education – also in translation
- HCP-patient Communication -
    31. HCP-patient Communication
- Other
    32. Other

Mechanisms

- Pain -
    1. Pain
- Trauma -
    2. General
    3. Prevention trauma related injury
- Fractures -
    4. Mechanism of fracture risk
    5. Fracture Healing
- Causes/risk factors -
    6. General
    7. Specific disease
    8. Modifiable Risk factors
- Prognosis/ predicting progression - *also in diagnosis*
    9. Prognosis/ predicting progression - *also in diagnosis*
- Prevention – also in diagnosis
    10. Prevention – *also in diagnosis*
- Understanding different phenotypes -
    12. Phenotypes general
    13. Personalised/stratified care- *also in diagnosis*

14. Role of Inflammation
15. Nutrition
16. Degeneration/Ageing
17. Repair/Regeneration
18. Lifecourse
19. Biomarkers - also in translation

Hereditary/Genetic influences - *also in translation -*

20. Genetics general
21. Role of sex Hormones
22. Ethnicity

Disease interactions -

23. MSK
24. Comorbidities
25. Treatment-related mechanisms
26. Microbiome
27. Tissue and Molecular Mechanisms
28. Other

Translation

Early Translation

1. Non specific
2. Devices
3. Cells
4. Genetics
5. Biomarkers
6. Technology
7. other

Late Translation

8. Models of care – *similar to optimal care pathways in living well*

Knowledge mobilisation (KMb) -

9. Role of KMB
10. *10.* HCP education *also in living well*
11. Lay dissemination
12. Service user involvement In KMB

Implementation -

13. Researching barriers to implementing evidence based practice
14. Ensuring delivery of standardised EBP (reducing inequalities – *also in living well)*
15. Improving/enhancing Implementation of EBP/guidelines
16. Systems to support implementation
17. Outcomes

18. Policy
19. Technology/Digital innovation
20. Other

5. Themes from subgroup review

Diagnosis

Opportunities for EHR/big data

1. Machine learning/artificial intelligence
2. New approaches for collecting routine data
3. Patient held EHR and linkage
4. Using EHR for prediction/personalised care

Early diagnosis

5. Optimising timely/prompt diagnosis
6. Value of early diagnosis to individual

Improving diagnosis

7. Refining diagnosis and definitions
8. Tools, tests and biomarkers to support diagnosis

Barriers to diagnosis and referral

9. Barriers to diagnosis and referral

Personalised/stratified care

10. Prediction models

Impact

11. In general/to society
12. To individual
13. True cost

Prognosis

14. Prognosis

Prevention

15. Prevention

Public health

16. Screening
17. Public Health messaging

Living well

1 Healthcare access provision and delivery	1.1 Improving access
	1.2 Provision of care - Optimal care pathways
	1.3 Delivery of Standardised care
	1.4 Addressing inequalities
2 Personalised bespoke approaches to care	
3 Self-management	3.1 Supporting self-management (including through networks)
4 Healthcare professional development	4.1 HCP Education
	4.2 HCP-patient communication
5 Treatments	5.1 New treatments
	5.2 Optimising combinations of treatments
	**5.3 Optimising specific treatments**
	5.3.1 Optimising pharmaceutical treatments
	5.3.2 Optimising lifestyle interventions including nutrition
	5.3.3 Optimising aids and devices
	5.3.4 Optimising exercise
	5.3.5 Optimising psychological support
	5.3.6 Optimising surgery
	5.4 Supporting behaviour change and adherence
	5.5 Monitoring and review
	5.6 Symptom management and quality of life (including pain)

Mechanisms

1. Pathological damage and pain
2. Pathological damage and pain chronicity
3. Pain pathways
4. Risk Factors: Genetics and Ethnicity
5. Sex Hormones
6. Life course and Aging
7. Impact of injury on MSK
8. Risk Factors – Nutrition & Microbiome
9. Lifestyle
10. Predicting cause of disease
11. Disease and molecular classification
12. Patient stratification
13. Biomarkers
14. Tissue and Molecular Mechanisms
15. Role of inflammation
16. Disease interactions & Co-morbidities
17. Drug development and management
18. Regeneration / Repair mechanisms
19. Prevention

Translation

1. Identifying and overcoming barriers to implementation
2. Knowledge translation/mobilisation
3. Policy/ Support for implementation
4. Timeliness and importance of translation across pipeline
5. Overcoming barriers to translation in research
6. Improving partnership
7. Granular discovery questions - test/monitoring
8. Granular discovery questions – interventions

6. Researcher breakdown results from E-survey 2

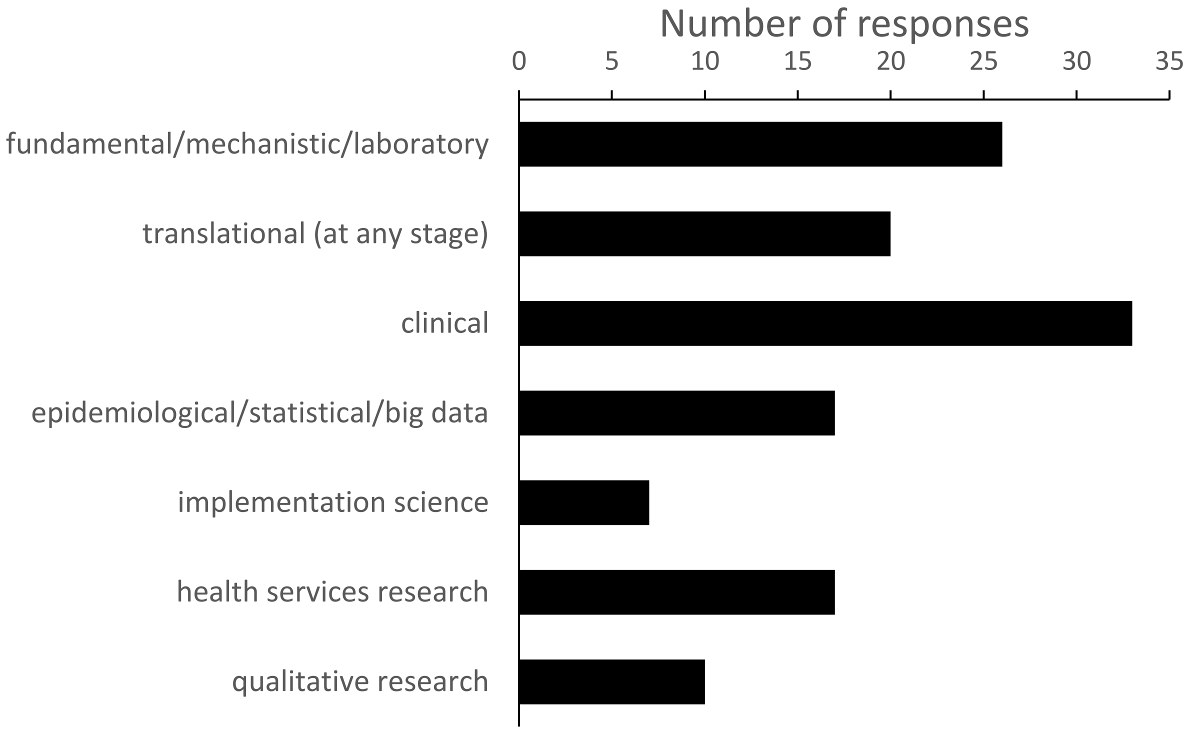

Those respondents who identified themselves as researchers (clinical or non-clinical) were asked to select the type of research they carry out. Respondents could select more than one option and this graph shows the number of times each type was selected in total.

7. Data completeness and Median scores by responder characteristics

Research avenue and criterion		Number who responded to the question with a score	Number of skipped fields (missing data)	Number of 'Unsure' responses	Median Scores (Lower quartile – Upper quartile)					
					Overall	Lay	Healthcare Professional	Researcher (clinical and non-clinical)	Complete responders	Partial responders
Test artificial intelligence approaches to analyse electronic health records and other large databases, (e.g. of x-rays or scans) to improve care.	Importance	213	3	15	7 (5-8)	7.5 (6-9)	6 (5-7)	7 (5-8)	7 (5-8)	7 (5-9)
	Impact	206	5	20	7 (5-9)	7 (6-9)	7 (3.5-8)	7 (5-8)	7 (5-8)	8 (5-9)
Study whether new ways of collecting and using standard health data will improve people's care and help society.	Importance	210	3	18	6 (4-8)	7 (5-8)	5 (4-7.25)	6 (3-7)	6 (4-8)	6 (3-8)
	Impact	204	6	21	6 (4-8)	7 (5-8)	5 (3-7)	5.5 (3.75-7.25)	6 (4-8)	6 (3-8)
Set up and test whether patients holding their own health data records helps them better manage their condition and make	Importance	215	2	12	7 (4-8)	8 (6-9)	7 (4-7)	5 (3-6.25)	6 (4-8)	8 (5-8.25)
	Impact	213	2	14	7 (5-8)	7 (6-9)	6 (3.8)	5 (3-7)	6 (5-8)	8 (6-9)

decisions with their clinician.										
Set up and test new ways of using electronic health records for accurate, earlier diagnosis and personalised monitoring.	Importance	213	3	14	7 (5-9)	8 (6-9.25)	6 (3-8)	6 (4.25-8)	7 (5-9)	7 (5-8)
	Impact	212	4	14	7 (5-9)	7.5 (6-9)	6 (3-8)	7 (5-8)	7 (5-9)	8 (6.75-9)
Find out how to improve accurate and earlier diagnosis of MSK conditions.	Importance	218	3	10	8 (6-9.25)	8 (7-10)	7 (5.5-8)	7 (5.5-9)	8 (6-9)	8 (6-10)
	Impact	219	5	7	8 (6-10)	9 (7-10)	7 (5-9	7 (5-9)	8 (6-10)	8 (6.25-10)
Understand how having an early diagnosis affects people, healthcare, and society.	Importance	222	3	5	7 (5-9)	8 (6-9)	6 (5-8)	5 (4-7)	7 (5-9)	7 (5-9.75)
	Impact	219	6	5	7 (5-9)	8 (6-9)	6.5 (5-8	6 (5-7)	7 (5-9)	7 (5-10)
Understand the best ways to diagnose and describe MSK conditions. Define features of relevant smaller groups within the same condition which make a difference to outcomes or care.	Importance	214	3	13	7 (5-8)	7 (6-9)	6 (4.5-7.5)	6 (5-8)	7 (5-8)	7.5 (5.75-9)
	Impact	214	3	13	7 (5-8)	8 (6-9)	6 (4-8)	6 (5-8)	7 (5-8)	8 (6-9)

Test how clinical tools, tests and markers can improve diagnosis.	Importance	217	3	11	7 (6-8.5)	8 (7-9)	6 (5-7.75)	7 (6-8)	7 (6-8)	7.5 (6-9)
	Impact	216	3	12	7 (6-9)	8 (6.75-9.25)	6.5 (5-8)	7 (5-8)	7 (6-9)	7.5 (5.75-9)
Understand the reasons why diagnosis is sometimes delayed, and how best to reduce delays.	Importance	223	3	4	7 (5-9)	8 (6.5-9)	6 (4-8)	6 (5-7)	7 (5-8)	7 (5-10)
	Impact	222	3	5	7 (5-9)	8 (6-10)	7 (5-8)	6 (5-8)	7 (5-9)	8 (6.75-10)
Test new ways of making sure the right person gets the right treatment.	Importance	219	2	10	8 (6-9)	8(6.25-10)	7 (5-8)	7 (5.5-8)	8 (6-9)	8 (7-9)
	Impact	217	4	10	8 (6-9)	8 (7-10)	7 (5-8)	7 (6-8.5)	8 (6-9)	8 (6.25-9)
Understand the links between MSK and other long-term conditions, and the effect they have on people, work and society.	Importance	220	3	7	7 (6-9)	8 (7-9)	7 (4-8)	7 (5-8)	7 (6-9)	8 (6-9)
	Impact	215	4	11	7 (5-9)	8 (7-9)	6 (5-8)	6 (5-8)	7 (5-9)	7 (6-9)
Study the best ways to measure the true effects of MSK conditions on individuals.	Importance	218	3	9	7 (5-9)	8 (7-10)	6 (4-7.25)	6 (4-8)	7 (5-9)	7 (5.75-9)
	Impact	215	6	9	7 (5-8)	8 (7-9.25)	6 (4-7)	6 (4-7.25)	7 (5-8)	7 (5-9)
Find out how to produce a better estimate of the true cost of long-term MSK	Importance	214	4	12	7 (5-8)	7 (6-9)	6 (4-8)	5.5 (4-7)	7 (5-8)	7 (5.75-9.25)
	Impact	208	7	15	6 (5-8)	7 (5-9)	6 (3-7.25)	5 (3-7)	6 (5-8)	6 (4.5-9)

conditions to people and society.										
Identify any groups of patients or patterns within a condition which inform on the course or outcomes of MSK conditions.	Importance	214	3	12	7 (5-8)	8 (6-9)	6 (5-8)	7 (5-8)	7 (5-8)	7 (6-8)
	Impact	212	4	13	7 (5-8)	7 (6-9)	6 (4.5-7.25)	7 (5-8)	7 (5-8)	7 (6-9)
Study if making changes to risk factors can prevent or delay the start of MSK conditions.	Importance	222	2	6	7 (6-9)	8 (7-9)	7 (5-8)	7 (6-8)	7 (6-9)	8 (6-10)
	Impact	217	6	7	7 (5-9)	8 (6-9)	7 (5-8)	7 (5-8)	7 (5-9)	8 (5-8.5)
Define the risk factors in MSK conditions that might predict important outcomes or enable screening.	Importance	216	3	12	7 (6-8)	8 (7-9)	6 (5-7.5)	7 (6-8)	7 (6-8)	8 (6-9)
	Impact	213	4	14	7 (6-9)	8 (7-9)	6 (5-8)	7 (6-8)	7 (6-8)	7 (5-9)
Study the pros and cons of screening for MSK conditions for people, healthcare and society.	Importance	215	4	12	6 (5-8)	7 (5-8.75)	6 (5-7)	6 (3-7)	6 (5-8)	7 (5-8.25)
	Impact	212	5	14	6 (4-8)	7 (5-8.75)	6 (4-7)	6 (3.5-7)	6 (4-8)	7 (5-9)
Study whether increasing public awareness of MSK conditions will encourage people to	Importance	222	2	6	6 (4-8)	7 (5-8)	6 (4-8)	6 (4-7.75)	6 (4-8)	7 (4-8)
	Impact	216	5	9	6 (4.25-8)	7 (5-8)	6 (3.75-8)	6 (4-8)	6 (4.75-8)	6 (4-8.25)

have a healthier lifestyle, get an earlier diagnosis and better care.										
Understand how changes in society, work and people's circumstances, including finances, might lower the risk or effects of MSK conditions.	Importance	215	1	14	7 (4-8)	7 (5-9)	6 (4-8)	6 (4-7)	7 (4-8)	7 (4-8)
	Impact	210	3	17	6 (4-8)	6 (5-9)	6 (3-7.5)	6 (4-7)	6 (4-8)	7 (4.5-8)
Understand the links between tissue damage and pain.	Importance	217	4	10	8 (6-9.5)	9 (7-10)	7 (6-8)	7 (5-9)	8 (6-9.75)	8 (7-9.5)
	Impact	219	6	6	8 (6-10)	9 (7.75-10)	7 (5-8)	7 (5-9)	8 (6-10)	8 (5.5-9.5)
Study how chronic pain develops.	Importance	222	5	4	8 (7-10)	9 (8-10)	7 (5-9)	8 (6-9)	8 (7-9.75)	8 (7-10)
	Impact	220	7	4	8 (6-10)	8 (7-10)	7 (5-9)	8 (5.75-9)	8 (6-9)	9 (6.5-10)
Understand why people's pain experiences are different and why some people develop chronic pain when others do not.	Importance	227	2	1	8 (7-10)	9 (7-10)	7 (5.25-9)	8 (7-9)	8 (7-9)	8 (7-10)
	Impact	225	2	3	8 (7-9)	9 (7-10)	7 (5-9)	8 (6-9)	8 (7-9)	8 (6-10)
Study how genes or ethnicity affect how MSK conditions develop.	Importance	219	4	8	8 (6-9)	8 (7-10)	7 (5-9)	7 (6-8)	8 (6-9)	8 (7-9)
	Impact	216	4	11	7 (5-9)	8 (6-10)	7 (4.75-8.25)	6 (4-7)	7 (5-9)	7 (5-9)

Understand how sex hormones and menopause change the risk of MSK conditions.	Importance	215	4	12	8 (6-9)	8 (7-10)	7 (5.5-8.5)	7 (6-8)	8 (6-9)	8 (7-10)
	Impact	215	4	12	7 (6-9)	8 (6-10)	7 (5-8.5)	7 (6-8)	7 (6-9)	8 (6-9.25)
Study biological disease processes to identify ways to predict if MSK conditions will develop or if existing conditions will progress during someone's lifetime.	Importance	213	5	12	8 (7-9)	8 (7-10)	7 (6-8)	8 (7-8)	8 (6.5-9)	8 (7.25-10)
	Impact	213	4	13	8 (6-9)	8 (7-9)	7 (5.75-8)	7 (5-8)	7 (6-9)	8 (6.25-10)
Study how injury can lead to an increased risk of developing MSK conditions.	Importance	220	2	9	8 (6-9)	8.5 (7-10)	7 (5.75-8)	7 (5-8)	8 (6-9)	8 (6-9.5)
	Impact	220	4	7	7 (5-9)	8 (6-10)	7 (5-9)	6 (5-8)	7 (5-9)	8 (6-10)
Study how diet and gut bacteria can change the risk of developing MSK conditions.	Importance	213	3	15	8 (6-9)	8 (7-10)	8 (6-9)	7 (5-8)	8 (6-9)	7 (6-9.5)
	Impact	213	3	15	7 (5-9)	8 (7-10)	8 (5-9.25)	6 (5-7.5)	7 (5-9)	7 (5-9.75)
Study the effect of lifestyle (e.g. work and exercise) on how MSK conditions develop and progress.	Importance	224	4	3	8 (6-9)	8 (7-10)	7 (6-9)	7 (6-8)	8 (6-9)	7.5 (6-9)
	Impact	223	4	4	8 (6-9)	8 (7-9)	7 (5-9)	7 (6-8)	8 (6-9)	7 (6-9)
	Importance	219	5	7	8 (6-9)	8 (7-10)	7 (6-8)	7 (6-9)	7 (6-9)	8 (7-10)

Identify how to earlier predict the progress of MSK conditions.	Impact	214	6	11	8 (6-10)	8 (7-10)	7 (5-9)	8 (6-9)	8 (6-9)	8 (7-10)
Understand disease processes so that we can better identify differences (subgroups of people) within the same condition.	Importance	218	7	5	8 (7-9)	8 (7-9)	7 (6-8)	8 (7-10)	8 (7-9)	8 (7-9)
	Impact	219	6	5	8 (6-9)	8 (6-9)	7 (6-8)	8 (6-10)	8 (6-9)	8 (6-9)
Identify disease processes that will allow better targeting of treatment to improve people's outcome.	Importance	217	5	7	8 (7-10)	8 (7-10)	7 (6-9)	8 (7-10)	8 (7-10)	8 (7-9.75)
	Impact	217	6	6	8 (7-10)	8 (7-10)	8 (6-9)	8 (7-9.75)	8 (7-10)	8 (7-9.75)
Identify tools, tests and markers that can diagnose disease at an early stage and inform whether the disease will progress and respond to treatment.	Importance	221	6	5	8 (7-10)	8 (7-10)	7 (6-9)	8 (7-9)	8 (7-10)	8 (7-10)
	Impact	220	7	5	8 (6-10)	8 (7-10)	8 (6-9)	8 (6-10)	8 (6-10)	8 (6-9)
Understand how key biological disease processes drive development and progress of MSK conditions.	Importance	213	5	13	8 (6-9)	8 (7-9)	7 (6-8)	8 (7-9)	8 (6-9)	8 (6.25-9)
	Impact	209	8	14	7 (6-9)	8 (6.25-9)	7 (5-9)	7 (6-9)	7 (6-9)	8 (5.25-9)

Study the role of inflammation in 'non-inflammatory' MSK conditions.	Importance	208	4	19	8 (7-9)	8 (7-10)	7 (6-8)	7 (6-9)	8 (7-9)	8 (6-9)
	Impact	205	4	22	7 (6-9)	8 (7-9.25)	7 (5-8)	7 (5-8)	7 (6-9)	7 (6-8.75)
Understand the biological links between MSK conditions and other illnesses.	Importance	217	6	8	8 (7-9)	8 (7-10)	7 (6-8.25)	7 (6-8.5)	8 (7-9)	8 (7-9)
	Impact	216	8	7	7.5 (6-9)	8 (7-10)	7 (6-8.25)	6 (5-8)	7 (6-9)	8 (6-9.25)
Identify biological targets to develop new treatments which change the course of disease.	Importance	214	3	13	8 (7-10)	8 (7-10)	7 (6-9)	8 (7-9)	8 (7-10)	8 (7-10)
	Impact	212	5	13	8 (7-10)	8 (7.25-10)	8 (6-9)	8 (7-10)	8 (7-10)	8.5 (6.75-10)
Understand how MSK tissues repair themselves and how this could be enhanced to improve MSK conditions.	Importance	224	3	3	8 (7-10)	9 (8-10)	8 (7-9)	8 (6.25-9)	8 (7-10)	9 (7-10)
	Impact	225	3	2	8 (6-10)	9 (8-10)	7 (6-9)	7.5 (5.25-9)	8 (6-10)	9 (7-10)
Study whether a better understanding of disease processes can be used to develop new ways of preventing MSK conditions.	Importance	218	5	8	8 (7-9)	8 (7-10)	7 (6-8.75)	8 (7-9)	8 (7-9)	8 (7-9)
	Impact	213	8	10	8 (6-9)	8 (7-10)	7 (5-9)	8 (6-9)	8 (6-9)	8 (7-9)
	Importance	207	7	9	7 (5-9)	8 (5-9)	6 (3-8)	5 (3-6.5)	6 (4-8)	8 (6-9)

Understand and address the reasons why people have difficulty or delays accessing care.	Impact	204	7	12	7 (5-9)	7 (6-9.25)	7 (4.25-8.75)	6 (5-7)	7 (5-8.75)	7.5 (6.25-9)
Decide the best ways of delivering remote care for people.	Importance	203	6	16	7 (4-8)	7 (5-8)	6 (3.5-8)	6 (4-7)	7 (4-8)	8 (6-9)
	Impact	201	9	15	7 (5-8)	7 (5-8)	6 (4.5-8)	7 (5-8)	7 (5-8)	8 (6-8)
Understand and address the reasons why everyone with MSK conditions does not receive minimum standards of care.	Importance	212	5	9	7 (5-9)	9 (7-9.75)	6 (5-8)	5 (3.25-7)	7 (5-9)	8 (6-9)
	Impact	209	7	10	7 (5-9)	8 (6-9)	7 (5-9)	6 (4-8)	7 (5-9)	7 (5.5-10)
Understand and address the reasons why everyone with a particular MSK condition does not have the same access to care.	Importance	204	7	13	7 (5-8.75)	8 (6-9)	6 (3.75-8)	5 (3-7)	7 (4-8.75)	8 (6-8.75)
	Impact	203	5	16	7 (5-9)	8 (6-9.75)	6 (3-8)	6 (4-8)	7 (5-9)	8 (6-9)
Develop and test ways to target and personalise treatments to each individual.	Importance	213	6	6	8 (7-9)	8 (7-9)	7 (6-9)	8 (6-9)	8 (7-9)	8 (7-10)
	Impact	212	8	5	8 (7-10)	8.5 (7-10)	7 (6-9)	8 (6-9)	8 (6-10)	8 (7-9)
Identify the best way to deliver the best support and	Importance	218	4	4	8 (6-9)	8 (7-9)	7.5 (5.75-9.25)	6 (4-8)	7.5 (5-9)	8 (6-9)
	Impact	218	4	4	8 (6-9)	8 (7-10)	8 (6.75-9)	7 (5-8)	8 (6-9)	8 (7-9)

information to help people effectively self-manage their condition.											
Identify and find ways to address gaps in healthcare professional knowledge about MSK conditions.	Importance	208	6	11	7 (5-9)	9 (7-10)	7 (5.25-9)	5 (3-7)	7 (5-9)	8 (6-9)
	Impact	206	7	12	8 (5-9)	8 (7-10)	7 (5-9)	7 (4-8)	8 (5-9)	8 (6-10)
Identify the best approaches to improving communication about MSK conditions between patients and their healthcare professionals.	Importance	216	5	4	7 (5-8)	8 (6-9)	7 (4.5-8)	5 (3-7)	7 (5-8)	7 (6-8.75)
	Impact	211	8	6	8 (6-9)	8 (6-10)	7 (5-8)	6 (5-8)	7.5 (6-9)	8 (6-9)
Develop and test new treatments to prevent or reduce progression of MSK conditions.	Importance	216	5	5	9 (7-10)	9 (8-10)	8 (7-9.25)	8 (7-10)	9 (7-10)	8 (8-10)
	Impact	213	7	6	9 (7-10)	9 (8-10)	8 (7-9)	8.5 (7-10)	9 (7-10)	8 (7-10)
Investigate how best to combine treatments.	Importance	204	7	14	8 (6-9)	8 (7-10)	7 (6-9)	7 (5-8)	8 (6-9)	8 (7-9)
	Impact	200	8	17	8 (6-9)	8 (7-10)	8 (6-9)	7 (5-8)	8 (6-9)	8 (6.75-9)
Better understand the benefit, safety and use	Importance	209	6	10	7 (5-9)	8 (7-9)	6 (5-8)	6 (4.5 -8)	7 (5-9)	8 (5-9)
	Impact	210	6	9	7 (5-9)	8 (7-9)	7 (5-8)	7 (5-8)	7 (5-9)	8 (5.5-9)

of existing medicines, including injections.										
Identify the best lifestyle interventions.	Importance	214	5	8	7 (5-9)	8 (6-9)	8 (5-9)	6 (4.75-8)	7 (5-9)	8 (6-9)
	Impact	215	4	8	7 (6-9)	8 (6-9)	8 (6-9)	7 (5-9)	8 (6-9)	7 (5-9)
Identify the best aids, supports and other devices to help people live well.	Importance	212	4	8	7 (5-8)	8 (6-9)	6 (5-8)	6 (5-7)	7 (5-8)	7 (5-8.5)
	Impact	213	6	5	7 (6-9)	8 (7-10)	6 (5-8.25)	7 (5-8)	7 (6-9)	7 (5-9)
Identify the surgical techniques, technologies and implants that help people the most.	Importance	206	6	13	8 (6-9)	8 (7-10)	6 (5-8)	7 (5-8)	8 (6-9)	8 (6-9)
	Impact	205	7	13	8 (6-9)	9 (7-10)	7 (5-8)	7 (6-8)	8 (6-9)	8 (5.75-9)
Find out more about the benefits, safety and best ways to use exercise and/or rehabilitation.	Importance	218	3	3	8 (6-9)	8 (7-9)	8 (6-9)	7 (5.25-8)	7 (6-9)	8 (7-10)
	Impact	215	4	5	8 (6-9)	8 (6-9)	8 (6-9)	7 (6-8)	8 (6-9)	8 (7-9.75)
Develop and test approaches to identify and help people with MSK conditions who need psychological support.	Importance	214	3	8	7 (6-8)	8 (7-9)	7 (5-9)	6 (5-7)	7 (6-8)	7 (6-9)
	Impact	212	3	10	8 (6-9)	8 (7-9)	7 (5.75-9)	7 (6-8)	8 (6-9)	8 (6-8.25)
	Importance	215	3	7	7 (6-9)	8 (7-9)	7 (6-8)	7 (5-8)	7 (6-9)	8 (7-9)

Identify the best ways to improve outcomes after surgery.	Impact	211	5	9	8 (6-9)	8 (7-9.75)	8 (6-9)	7 (5-8.25)	8 (6-9)	8 (7-9)
Develop and test approaches to help people with MSK conditions make lasting changes to improve their health.	Importance	217	4	4	8 (6-9)	8 (7-9)	8 (6-10)	6 (5-7)	7.5 (6-9)	8 (6-9)
	Impact	215	5	5	8 (6-9)	8 (7-9.25)	8 (7-9)	7 (5-8)	8 (6-9)	8 (7-8)
Understand and meet people's needs for monitoring and review of their condition.	Importance	207	6	10	7 (5-8)	8 (6.75-9)	7 (5-8)	6 (3-7)	7 (5-8)	7 (6-8)
	Impact	201	10	12	7 (5-8)	7 (6.5-9)	7.5 (5-8)	6 (4-8)	7 (5-8)	8 (6-8)
Identify the best ways to manage pain and/or improve quality of life.	Importance	220	4	3	8 (6-10)	9 (7-10)	7.5 (6-9)	7 (5-8)	8 (6-9.75)	8 (7-10)
	Impact	218	6	3	8.5 (7-10)	9 (7-10)	8 (7-9)	8 (6-9)	8 (7-10)	9 (7.25-10)
Understand and overcome the barriers preventing research-proven tests and treatments being put into practice.	Importance	206	5	10	7 (5.75-9)	8 (6-9.25)	7 (6-9)	6 (4-7)	7 (5-9)	8 (6.5-10)
	Impact	200	8	13	8 (7-9)	8 (7-10)	8 (6.25-9)	7 (6-8)	8 (6-9)	8.5 (7-10)
Understand the best ways of providing research-proven treatments to people,	Importance	205	6	11	8 (6-9)	9 (7-10)	7 (5-8)	6 (4-8)	7 (5-9)	8 (7-9)
	Impact	203	8	11	8 (7-9)	8.5 (7-10)	8 (5-8)	7 (6-8.25)	8 (6-9)	8 (8-10)

including where, when and by whom.										
Study the best way of sharing the results of research with clinicians, scientists, policy makers and people with MSK conditions.	Importance	211	6	6	7 (4-9)	8 (6-9)	6 (5-8)	4 (3-7)	7 (4-9)	8 (5.5-9)
	Impact	207	7	9	7 (5-9)	8 (6-9)	7 (5-8.25)	6 (4-8)	7 (5-9)	8 (7-10)
Find better ways to speed up the uptake of research results into treatment guidelines and policy.	Importance	211	4	8	7 (5-9)	8 (6-10)	7 (4-8.25)	5.5 (3-7)	7 (5-9)	8 (5-10)
	Impact	211	5	7	8 (6-10)	8 (7-10)	8 (5-9)	7.5 (6-8.25)	8 (6-9)	8 (7-10)
Investigate ways to speed up the process of turning scientific research findings into effective treatments.	Importance	211	5	6	7 (6-10)	8 (7-10)	8 (5-10)	6 (3.5-8)	7 (5-9.25)	8 (7-10)
	Impact	211	5	6	8 (6-10)	8 (7-10)	8 (5-9)	8 (6-9)	8 (6-10)	8 (7-10)
Develop better ways to overcome the known difficulties in the process of turning a possible effective treatment into a safe, licensed product.	Importance	208	5	9	7 (5-9)	8 (7-10)	6 (5-8)	6 (3-8)	7 (5-9)	7 (6-9.5)
	Impact	204	7	11	8 (6-9)	8 (7-10)	7 (5-8.75)	7 (6-8)	8 (6-9)	8 (7-10)
Study the best way of bringing together	Importance	211	6	5	7 (5-9)	8 (7-10)	7 (4-9)	6 (3-8)	7 (5-9)	8 (6.25-10)
	Impact	209	6	7	7 (5-9)	8 (7-10)	7 (4-9)	7 (4-8)	7 (5-9)	8 (6.25-10)

scientists, clinicians, industry, policy makers and people with MSK conditions to improve the development of early research towards better available treatments.										
Study how new clinical, biological, genetic and technology approaches can improve diagnosis.	Importance	208	6	9	8 (6-9)	8 (7-10)	7 (6-8.5)	8 (6-8.5)	8 (6-9)	9 (7-10)
	Impact	206	6	11	8 (6-9)	8 (7-9)	7 (5.5-8.5)	8 (6-9)	8 (6-9)	8 (6.25-10)
Study how new clinical, biological and genetic and technology approaches can monitor the effectiveness of treatments.	Importance	204	5	14	8 (6-9)	8 (7-10)	7 (6-8)	7 (5-8)	8 (6-9)	8 (6-10)
	Impact	200	7	16	8 (6-9)	8 (7-10)	7 (5-8)	7 (5-8)	8 (6-9)	7 (5-9)
Improve how information from lab-based research and clinical trials is used to safely speed up	Importance	208	6	9	7 (5-9)	8 (6.75-10)	7 (5.5-8.5)	6 (4-7)	7 (5-9)	7 (6-9)
	Impact	206	10	7	8 (6-9)	8 (7-10)	7 (5.5-9)	7 (5-8)	8 (6-9)	8 (6-10)

making the best treatments available.										

8. Median, means and distributions of each of the criterion scores for each research avenue

Supplementary Figure 8A: Box and Whisker plots indicating distribution for research avenue ranks 1-3: Research Avenue Scores (1-10) for: Important Knowledge (K) and Impact (I) shown as minimum, 25% quartile, median, 75% quartile and Maximum as box and whisker plots, mean values are overlayed (+) for each score. Research domains indicated in colours: Living well (Blue), Diagnosis (Purple), Mechanisms (Green), Translation (Peach). Within rank research avenues listed alphabetically.

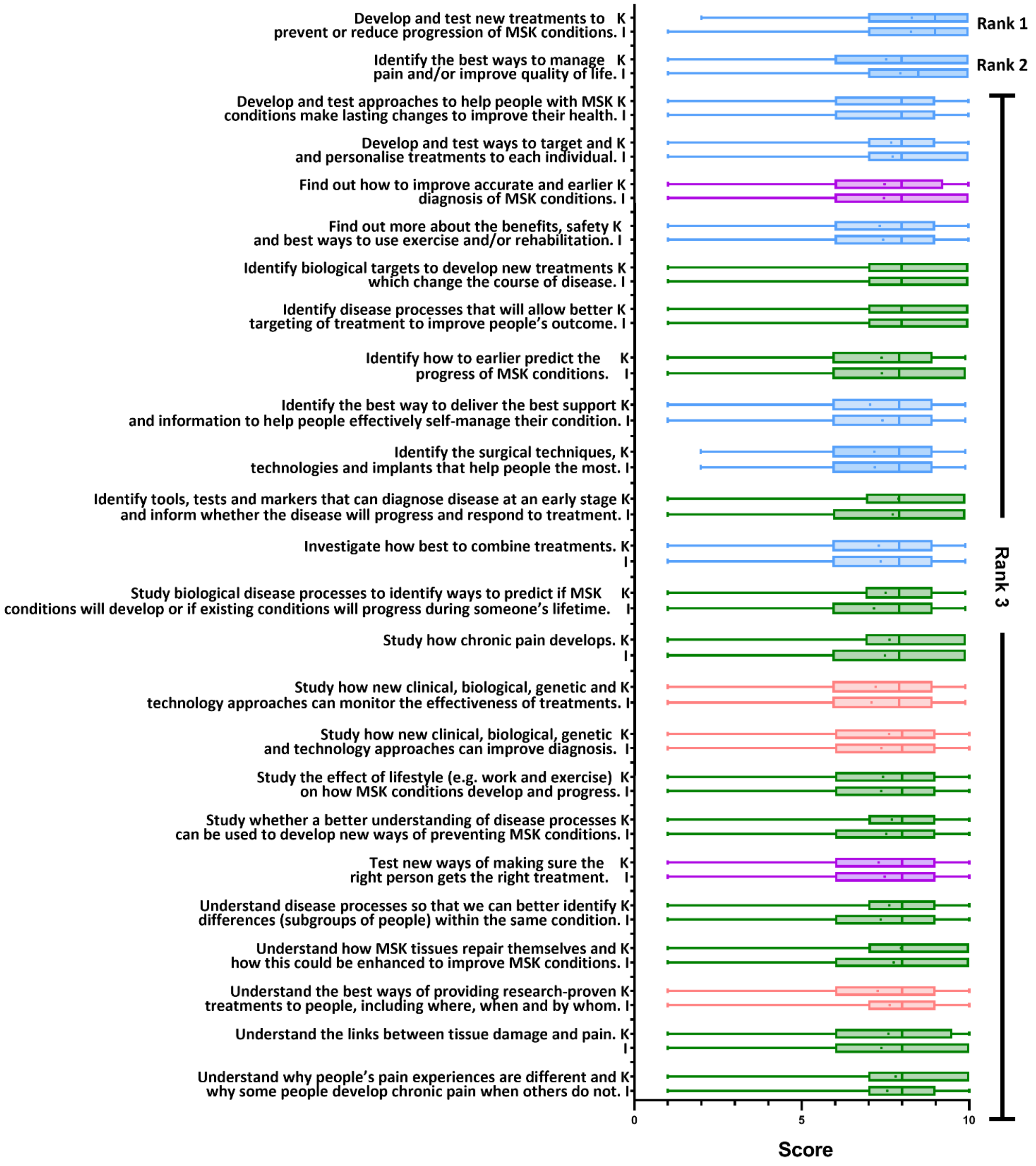

Supplementary Figure 8B: Box and Whisker plots indicating distribution for research avenue ranks 4&5: Research Avenue Scores (1-10) for: Important Knowledge (K) and Impact (I) shown as minimum, 25% quartile, median, 75% quartile and Maximum as box and whisker plots, mean values are overlayed (+) for each score. Research domains indicated in colours: Living well (Blue), Mechanisms (Green), Translation (Peach), note no avenues from diagnosis domain within ranks 4&5. Within rank research avenues listed alphabetically.

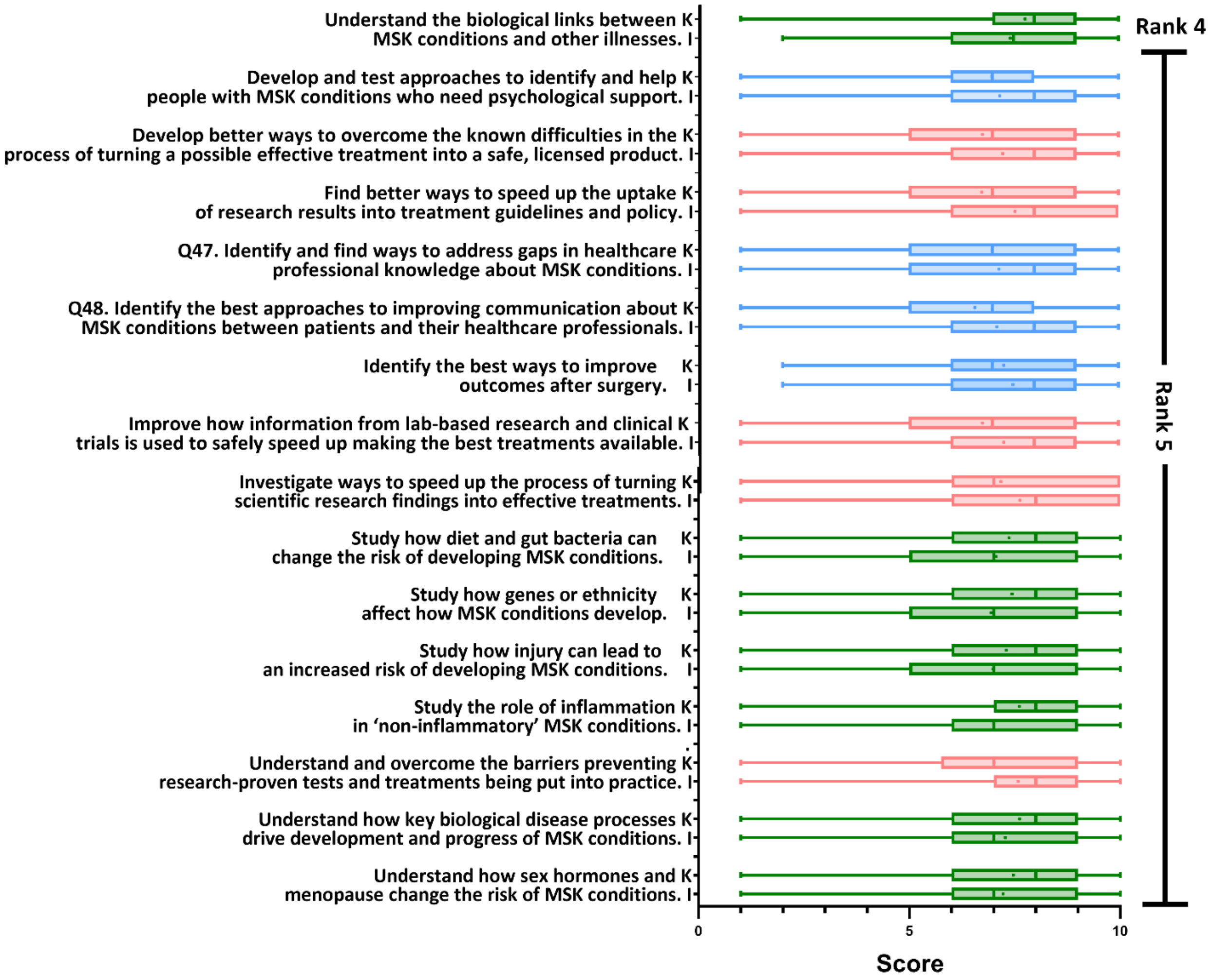

Supplementary Figure 8C: Box and Whisker plots indicating distribution for research avenue ranks 6-8: Research Avenue Scores (1-10) for: Important Knowledge (K) and Impact (I) shown as minimum, 25% quartile, median, 75% quartile and Maximum as box and whisker plots, mean values are overlayed (+) for each score. Research domains indicated in colours: Living well (Blue), Diagnosis (Purple), Translation (Peach), note no avenues from mechanisms domain within ranks 6-8. Within rank research avenues listed alphabetically.

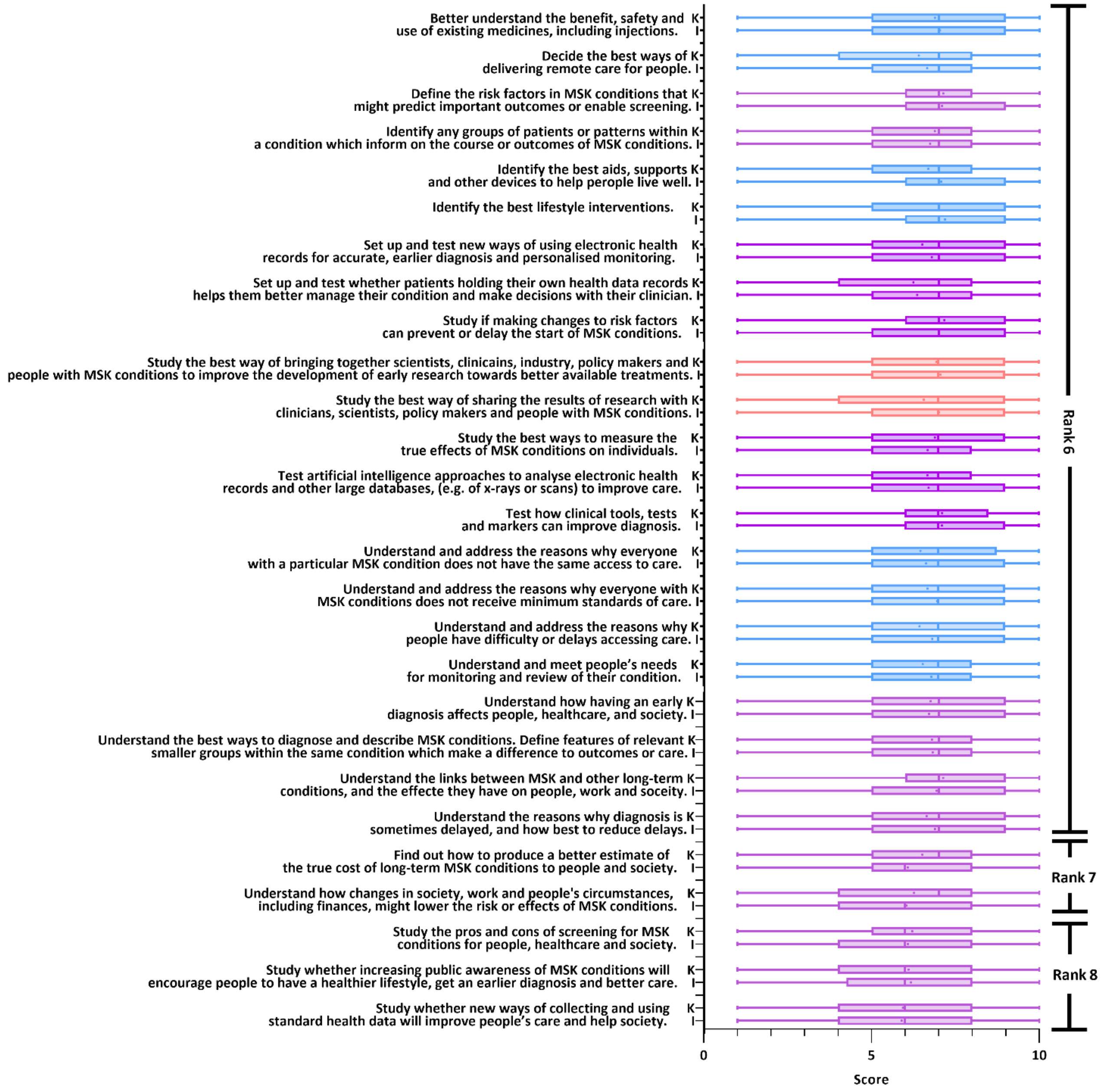

Supplement: Supplementary appendix 2 [file mmc2.pdf]
